# An exceptional fossil skull from South America and the origins of the archosauriform radiation

**DOI:** 10.1038/srep22817

**Published:** 2016-03-11

**Authors:** Felipe L. Pinheiro, Marco A. G. França, Marcel B. Lacerda, Richard J. Butler, Cesar L. Schultz

**Affiliations:** 1 Laboratório de Paleobiologia, Universidade Federal do Pampa, São Gabriel, Brazil; 2 Laboratório de Paleontologia e Evolução de Petrolina, Universidade Federal do Vale do São Francisco, Petrolina, Brazil; 3 Laboratório de Paleontologia de Vertebrados, Universidade Federal do Rio Grande do Sul, Porto Alegre, Brazil; 4 School of Geography, Earth & Environmental Sciences, University of Birmingham, Birmingham, UK

## Abstract

Birds, dinosaurs, crocodilians, pterosaurs and their close relatives form the highly diverse clade Archosauriformes. Archosauriforms have a deep evolutionary history, originating in the late Permian, prior to the end-Permian mass extinction, and radiating in the Triassic to dominate Mesozoic ecosystems. However, the origins of this clade and its extraordinarily successful body plan remain obscure. Here, we describe an exceptionally preserved fossil skull from the Lower Triassic of Brazil, representing a new species, *Teyujagua paradoxa*, transitional in morphology between archosauriforms and more primitive reptiles. This skull reveals for the first time the mosaic assembly of key features of the archosauriform skull, including the antorbital and mandibular fenestrae, serrated teeth, and closed lower temporal bar. Phylogenetic analysis recovers *Teyujagua* as the sister taxon to Archosauriformes, and is congruent with a two-phase model of early archosauriform evolution, in response to two mass extinctions occurring at the end of the Guadalupian and the Permian.

Birds, dinosaurs, crocodilians, and pterosaurs all belong to the clade Archosauriformes^1^, an extraordinarily diverse group that dominated terrestrial tetrapod faunas worldwide for nearly the entire Mesozoic Era^2^,^3^,^4^, around 175 million years, and plays a major role in the modern biota, with birds comprising around a third of extant tetrapod biodiversity^5^,^6^. The Permian origin of the clade and its major diversification during the Triassic following the end-Permian mass extinction event were events of exceptional significance that fundamentally reshaped ecosystems on land^2^,^7^,^8^,^9^,^10^,^11^. Several classic anatomical features relating to carnivorous adaptations and cranial pneumaticity characterize the archosauriform skull^1^,^8^,^11^. However, the acquisition of this highly successful cranial morphology from more primitive reptiles is poorly understood due to the patchy and fragmentary nature of the early archosauriform fossil record, and the absence of key transitional taxa showing intermediate morphologies^10^,^11^.

The oldest known archosauriforms consist of rare and highly fragmentary remains from the Permian of Russia^11^. Following the end-Permian mass extinction, c. 252 million years ago, fossils of archosauriforms and their nearest relatives become more common and globally distributed, but articulated specimens remain almost unknown outside a small number of well-sampled areas in South Africa and China^8^,^10^,^11^,^12^. Here, we report a new, exceptionally preserved skull from the Triassic of Brazil, which is the most complete tetrapod fossil yet discovered from the Lower Triassic of South America. This skull represents a previously unknown species that is the sister taxon to Archosauriformes and which fills a major morphological gap in understanding of early archosauriform evolution.

## Results

### Systematic palaeontology

Diapsida Osborn, 1903 *sensu* Laurin, 1991

Sauria McCartney, 1802 *sensu* Gauthier *et al.* 1988

Archosauromorpha Huene, 1946 *sensu* Gauthier *et al.* 1988

*Teyujagua paradoxa* gen. et sp. nov.

### Etymology

Genus named after Teyú Yaguá, one of the seven legendary beasts in the mythology of the Guarani ethnic group, who occupied a large territory of central east South America, including the type locality of the new species. Teyú Yaguá, literally meaning ‘fierce lizard’, is commonly represented as a dog-headed lizard. Species name derived from *paradoxa*, Greek meaning ‘paradoxical’, ‘unexpected’, owing to its unusual combination of plesiomorphic and derived characters.

### Holotype

UNIPAMPA 653, an almost complete, well-preserved skull with associated cervical vertebrae.

### Type locality and age

**Figure 1:**
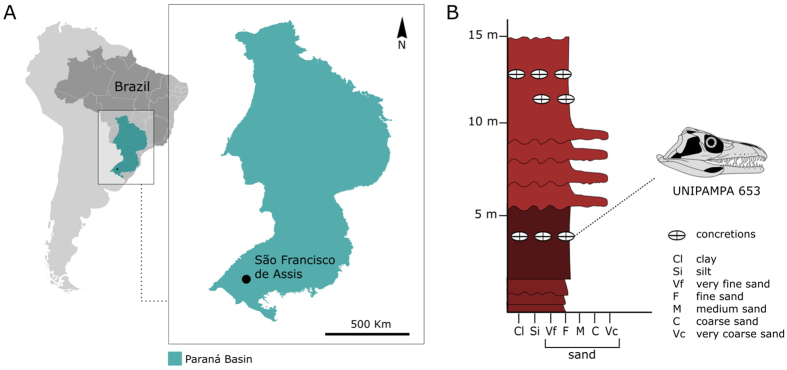
Type locality of *Teyujagua paradoxa*. (**A**), geographic map with the location of the Paraná Basin within Brazil and *Teyujagua* type location; (**B**), simplified stratigraphic profile of the outcrop, showing the level where *Teyujagua* was found. Area map was modified from Strapasson *et al.*^30^; stratigraphic profile modified from Da Rosa, *et al.*^13^.

Exposure of the Sanga do Cabral Formation^13^, Paraná Basin, São Francisco de Assis municipality, Rio Grande do Sul State, Southern Brazil (29°36′56″S, 55°03′10″W) (Fig. 1). An Induan to early Olenekian age (Lower Triassic) is inferred for the Sanga do Cabral Formation based on the presence of the parareptile *Procolophon trigoniceps*, and comparisons with the coeval *Lystrosaurus* Assemblage Zone of the South African Karoo^13^,^14^,^15^. *Teyujagua* was found in close association with archosauromorph vertebrae, cranial and postcranial remains of *P. trigoniceps*, temnospondyl amphibians and as-yet-unidentified tetrapod bones.

### Diagnosis

Archosauromorph with the following unique character combination: confluent, dorsally positioned external nares; maxilla participating in orbital margin; antorbital fenestra absent; trapezoidal infratemporal fenestra with incomplete lower temporal bar; teeth serrated on distal margins; surangular bearing a lateral shelf; external mandibular fenestrae present and positioned beneath the orbits when the lower jaw is in occlusion (autapomorphic for *Teyujagua*).

### Description

**Figure 2:**
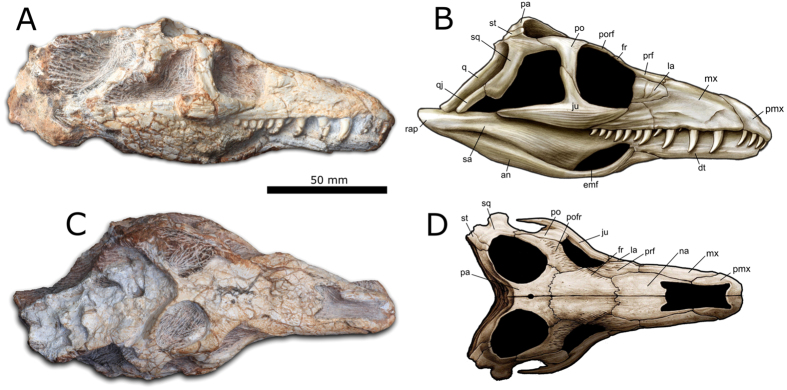
*Teyujagua paradoxa* holotype (UNIPAMPA 653). Photographs and interpretative drawings in right lateral (**A**,**B**) and dorsal (**C**,**D**) views. Abbreviations: an, angular; dt, dentary; emf, external mandibular fenestra; fr, frontal; ju, jugal; la, lacrimal; mx, maxilla; na, nasal; pa, parietal; pmx, premaxilla; po, postorbital; pofr, postfrontal; prf, prefrontal; q, quadrate; q j, quadratojugal; rap, retroarticular process; sa, surangular; sq, squamosal; st, supratemporal. Artwork by J. Anderson.

**Figure 3:**
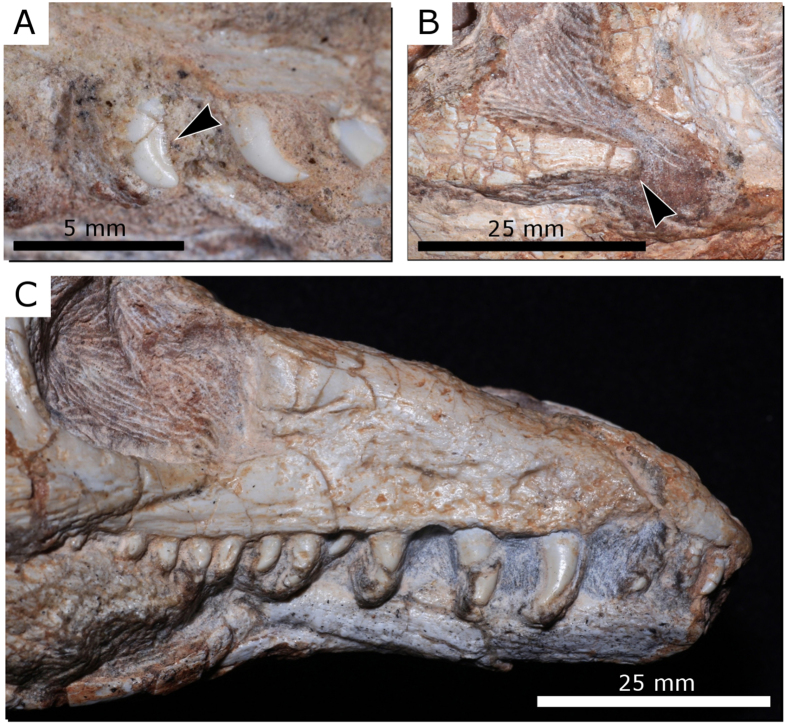
Close ups of the skull of *Teyujagua paradoxa* (UNIPAMPA 653). (**A**), posterior left maxillary teeth, showing serrations; (**B**), posterior process of the left jugal; (**C**), rostrum.

The 115 mm long skull is well preserved and almost complete, and is associated with four cervical vertebrae (Figs 2 and 3; Supplementary Fig. 1). The occipital and palatal regions and parts of the left side of the skull are still covered by the enclosing matrix, but were partially examined using computed tomography (CT) (Supplementary Fig. 2).

The snout is relatively broad and flattened, with dorsally positioned, confluent external nares. Dorsal confluent nares are an unusual condition that is often linked to aquatic or semi-aquatic habits, being present in many crocodyliforms, although they also occur in the terrestrial rhynchosaurs^16^, which are near relatives of early archosauriforms. The nasals contribute substantially to the skull table, followed by short and broad frontals, and parietals that bear a small pineal foramen. Although the loss of the pineal foramen has been identified as a synapomorphy of archosauriforms^8^, this structure is variably absent or present in the early archosauriform *Proterosuchus fergusi*^12^ and the close archosauriform relative *Prolacerta broomi*^17^. The prefrontals and sculptured postfrontals almost exclude the frontals from the dorsal orbital margin, whereas the maxilla participates in the anteroventral orbital margin. The orbits face anterolaterally, and were probably capable of at least limited binocular vision. The slender supratemporals are visible in dorsal view.

The premaxillae have well-developed, slender posterodorsal processes but lack anterodorsal processes, as a result of the confluent nares. An antorbital fossa/fenestra is absent from the maxillae (Fig. 3C). The jugals are triradiate, with main bodies ornamented with longitudinal ridges. The posterior jugal process tapers distally and does not reach the quadratojugal (Fig. 3B). The trapezoidal infratemporal openings were ventrally bordered by incomplete lower temporal bars, similar to the condition in non-archosauriform archosauromorphs such as *Prolacerta*, *Protorosaurus* and *Mesosuchus*^16^,^17^. By contrast, the lower temporal bar is complete in nearly all archosauriforms, although this character is variable in *Proterosuchus fergusi*^12^.

On the lower jaw, the surangulars bear lateral shelves that match closely with the ventral margins of the posterior processes of the jugals. The external mandibular fenestra is present, unusually anteriorly positioned, and ventrally bordered by a slender ascending process of the angular. The posterior contacts of the dentaries with the post-dentary bones cannot be identified. However, CT scans reveal that the dentary tooth row ends slightly anterior to the maxillary one (Supplementary Fig. 2).

Each premaxilla possesses four teeth and the maxilla had a maximum of 15. The teeth bear serrations on their distal margins only, as in proterosuchid archosauriforms, but differing from the condition in more derived archosauriforms in which serrations are usually present on both mesial and distal margins^18^ (Fig. 3A). Pronounced heterodonty is evident, with small premaxillary teeth followed by considerably larger anterior maxillary teeth. The teeth are labiolingually compressed, held in well-defined sockets, and not firmly associated with surrounding alveolar bone. Implementation therefore appears to be thecodont, rather than ankylothecodont as in many of the earliest archosaurifoms^19^.

### Phylogeny

**Figure 4:**
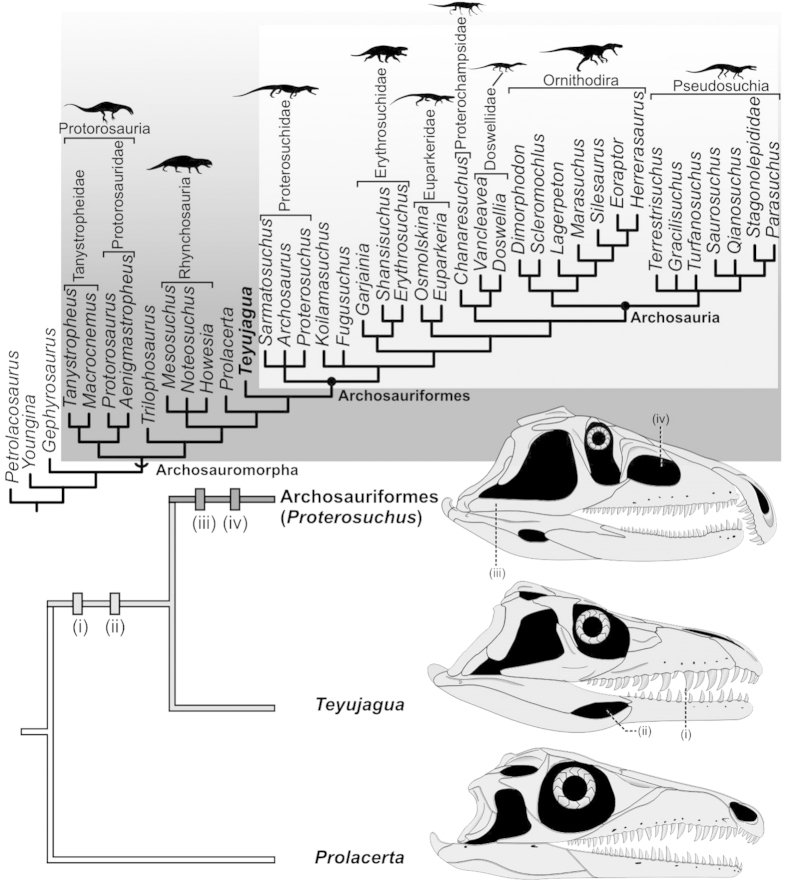
Archosauromorph phylogeny showing the recovered position of *Teyujagua.* Top: strict consensus tree summarising phylogenetic results. Bottom: sequence of acquisition of archosauriform features among the archosauromorphs *Prolacerta*, *Teyujagua* and the basal archosauriform *Proterosuchus*. (i) serrated teeth; (ii) external mandibular fenestra; (iii) closed lower temporal bar; (iv) antorbital fenestra. *Prolacerta* and *Proterosuchus* skulls redrawn from an artwork by M. Ezcurra. Not to scale.

Our novel cladistic analysis recovered two most parsimonious trees with 872 steps (Supplementary Fig. 3). The strict consensus of these topologies (Fig. 4) positions *Teyujagua* as the sister taxon of Archosauriformes, a position previously occupied by the Lower Triassic *Prolacerta*^8^,^11^,^17^. The clade *Teyujagua* plus Archosauriformes is supported by five synapomorphies: (i) Serrations on tooth crowns; (ii) trapezoidal shape of the infratemporal fenestrae; (iii) frontal-parietal suture at right angle to parasagittal plane; (iv) mandible bearing an external fenestra; (v) lateral shelf on surangular.

Archosauriformes includes the traditional basal groups, such as Proterosuchidae and Erythrosuchidae, together with the crown group Archosauria. Proterosuchidae consists of *Proterosuchus*, *Archosaurus* and *Sarmatosuchus*, although the relationships within this clade are unresolved. *Fugusuchus*, *Koilamasuchus* and the clade *Erythrosuchus* + Archosauria also have unresolved positions relative to one another. The clade *Chanaresuchus* + (*Doswellia* + *Vancleavea*) is recovered as the sister group of Archosauria, and Euparkeriidae is the sister group of this less inclusive clade. As such, four recognised groups compose non-archosaurian Archosauriformes: Proterosuchidae, Erythrosuchidae, Euparkeriidae and the clade including *Chanaresuchus* + (*Doswellia* + *Vancleavea*).

Another analysis was performed including the poorly known *Eorasaurus*, which may be the oldest known archosauriform. The analysis recovered 14 most parsimonious trees with 873 steps. Most of the recovered topologies are similar to those recovered in the first phylogenetic analysis. The consensus tree differs in positioning *Eorasaurus* in an unresolved polytomy together with *Koilamasuchus*, *Fugusuchus*, erythrosuchid taxa, and a clade composed of Euparkeriidae + Proterochampsia + Archosauria (Supplementary Fig. 5). This provides additional support for the archosauriform affinities of *Eorasaurus*, and the existence of archosauriform ghost lineages extending into at least the middle Wuchiapingian^11^.

## Discussion

*Teyujagua* presents an unexpected combination of basal archosauromorph and typical archosauriform features. For example, *Teyujagua* resembles basal archosauromorphs in lacking an antorbital fenestra and retaining open lower temporal bars^1^,^8^,^11^,^20^. However, *Teyujagua* possesses external mandibular fenestrae and serrated teeth, features previously considered unique to Archosauriformes^8^,^11^. Comparisons between *Prolacerta*, *Teyujagua* and early archosauriforms demonstrate for the first time that these key anatomical features of Archosauriformes were acquired in a mosaic fashion (Fig. 4). Serrated teeth and external mandibular fenestrae, important features underpinning the evolution of large, powerful hypercarnivores, were acquired before a closed lower temporal bar and the antorbital opening. Thus, key dietary adaptations emerged in the early history of the Archosauriformes before the onset of the major skull pneumatisation that played an important role in the group’s later evolutionary history^21^.

**Figure 5:**
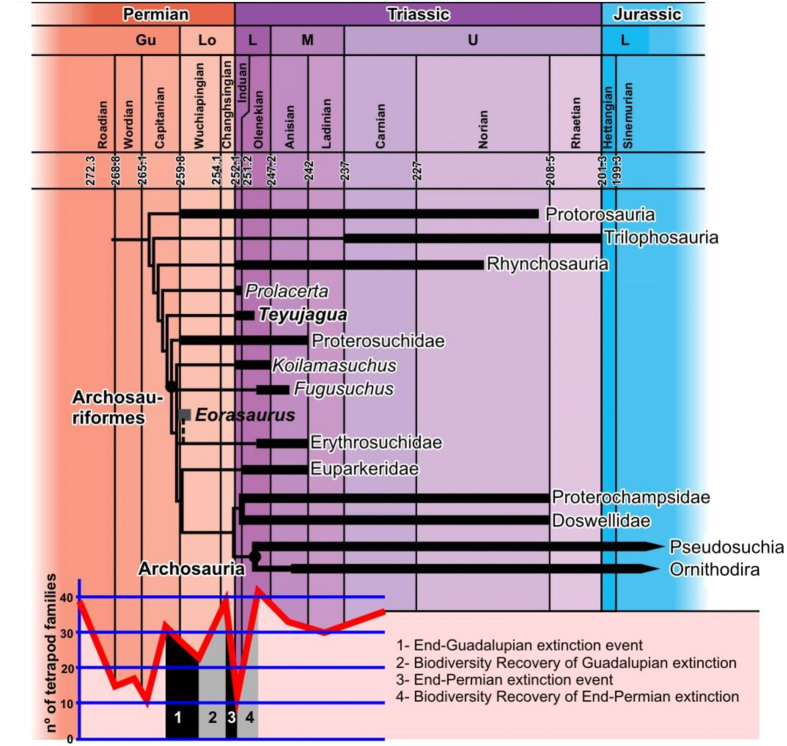
Simplified temporally calibrated phylogeny of Archosauriformes and close relatives (Archosauromorpha). A substantial diversification of archosauriforms and close relatives occurred in the late Permian (Lopingian), following the end-Guadalupian mass extinction, with a second radiation immediately following the end-Permian crisis. Diversity curve from Sahney and Benton^22^.

The oldest known unambiguous archosauriform is *Archosaurus rossicus*, from the uppermost Permian of Russia^11^,^19^. However, the late Guadalupian to early Lopingian age of the possible archosauriform *Eorasaurus*^11^ suggests that the clade had a substantial evolutionary history before the end-Permian mass extinction. Although hampered by the scarcity of Permian archosauromorph remains, the results of our phylogenetic analysis are congruent with two major pulses of opportunistic radiation experienced by early archosauriforms and close relatives such as *Teyujagua*. The first of these would be a phylogenetic diversification during the Lopingian, coincident with the recovery from the end-Guadalupian mass extinction^22^,^23^ (Fig. 5), perhaps as disaster taxa filling empty niches of small piscivores and predators. The rarity of archosauriform body fossils in Permian strata suggests that the clade formed a minor component in latest Palaeozoic faunas, when terrestrial trophic chains had therapsids as the main higher-level predators^7^,^22^,^24^.The end-Permian extinction disrupted these food chains, and during the faunal recovery the Archosauriformes underwent a major increase in abundance, size and species richness^7^,^19^,^22^,^24^, becoming the main terrestrial predators^7^,^22^,^24^, and later expanding to also dominate large herbivorous niches^8^,^9^. The ichnological record provides additional support for this two-phase radiation^25^. The discovery of *Teyujagua* thus helps to clarify early archosauriform evolution, allowing a better understanding of how this clade rose to dominate Mesozoic faunas and shape the modern biota.

## Methods

### Type horizon and locality

The type locality of *Teyujagua paradoxa* is a known fossil site that has been thoroughly described by Da Rosa *et al.*^13^. The locality is informally known as Bica São Tomé and is situated about 10 km east of São Francisco de Assis municipality, Rio Grande do Sul State, Southern Brazil. *Teyujagua* was found in one of the five outcrops that compose the Bica São Tomé (outcrop 5 of Da Rosa *et al.*^13^). The outcrop consists of a 15 m thick section with a predominance of fine reddish sandstones, intercalated with coarse sandstones and intraformational conglomerates, this being a typical lithology of the Sanga do Cabral Formation^13^. The *Teyujagua* holotype was found in a layer rich in calcareous concretions, about 5 m from the baseline of the outcrop. The fossil assemblage so far reported for the Bica São Tomé site is dominated by procolophonoid cranial and postcranial material, some of which is referable to *Procolophon trigoniceps*. Less commonly, the site has produced temnospondyl cranial and postcranial fragments, incomplete long bones attributable to Cynodontia and incomplete archosauromorph vertebrae. Sanga do Cabral Formation fossils are typically found in association with the intraformational conglomerates and, as a result, show signs of intense reworking and fragmentation. The holotype of *Teyujagua paradoxa* is the most complete vertebrate specimen collected in this sedimentary unit to date. An Induan to early Olenekian (Lower Triassic) age is inferred for the Sanga do Cabral Formation based on the presence of *Procolophon trigoniceps*, which is restricted to the upper Katberg Formation (*Lystrosaurus* Assemblage Zone) in the Karoo Basin of South Africa^13^,^14^,^15^. *Procolophon trigoniceps* is the only taxon from the Sanga do Cabral Formation to provide biostratigraphic correlations with other Lower Triassic sedimentary units. Notably, in the South African Karoo Basin the oldest remains of *Procolophon trigoniceps* are found 116 m above the Permo-Triassic boundary^24^.

### Phylogenetic analysis

In order to test the phylogenetic position of *Teyujagua*, a novel data matrix was assembled including taxa and morphological characters from two previous studies that aimed to address the phylogeny of Archosauromorpha and that of basal archosauriforms outside Archosauria^11^,^26^. In total, the dataset included 44 operational taxonomic units (OTUs) and 252 morphological characters (see the supplementary material for full details).

The analysis was performed using TNT version 1.1^27^. *Petrolacosaurus* was considered the outgroup taxon and all characters were treated with equal weight. Of the 252 characters, 205 are binary and 47 are multistate. The following 35 multistate characters were *a priori* considered as ordered: 18, 19, 20, 21, 26, 33, 39, 42, 43, 55, 62, 64, 65, 69, 74, 81, 103, 113, 118, 127, 144, 148, 149, 155, 159, 165, 167, 170, 173, 177, 193, 206, 218, 231, 238.

The analysis consisted of a heuristic search of 1000 replicates using random addition sequences followed by tree-bisection-reconnection (TBR) branch swapping, retaining ten trees per replicate, with branches not supported by at least one synapomorphy collapsed following the search. If some replications overflowed during the first round of analysis, a traditional search was again employed, this time using trees from RAM. Bremer and Bootstrap supports were obtained following the analysis^28^,^29^ (Supplementary Fig. 4).

## Additional Information

**How to cite this article**: Pinheiro, F. L. *et al.* An exceptional fossil skull from South America and the origins of the archosauriform radiation. *Sci. Rep.* **6**, 22817; doi: 10.1038/srep22817 (2016).

## Supplementary Material

Supplementary Information
