# Supplementary material for: An exceptional fossil skull from South America and the origins of the archosauriform radiation
Source: Sci Rep. 2016 Mar 11;6:22817. doi: 10.1038/srep22817 (PMC4786805; doi:10.1038/srep22817)
Supplement: Supplementary Information [file srep22817-s1.doc]

An exceptional fossil skull from South America and the origins of the archosauriform radiation

Felipe L. Pinheiro, Marco A. G. De França, Marcel B. Lacerda, Richard J. Butler, Cesar L. Schultz

**Supplementary Figures**


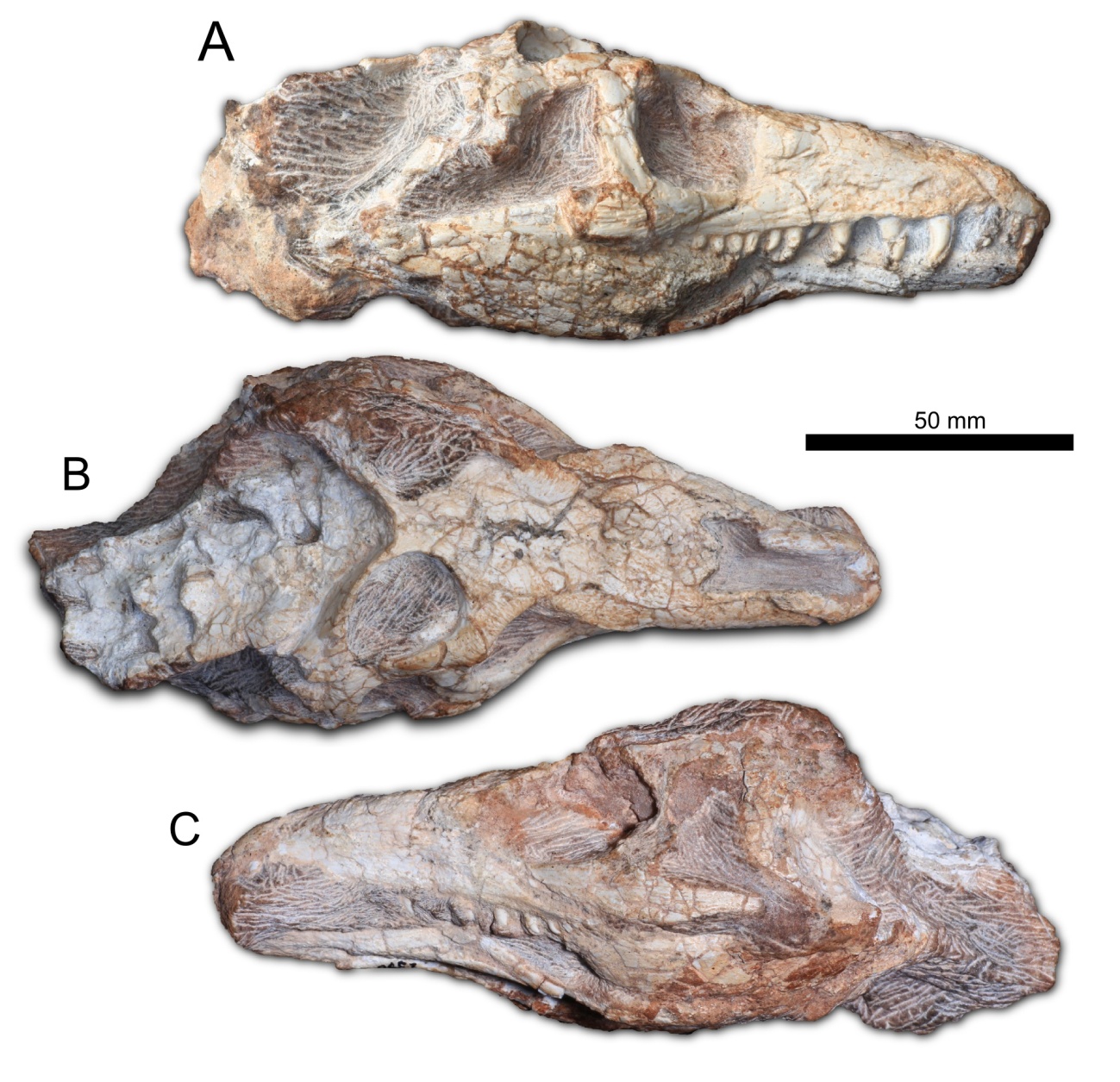


**Supplementary Figure 1.** **Photographs of the skull of *Teyujagua paradoxa* (UNIPAMPA 653).** A, right lateral view; B, dorsal view; C, left lateral view.


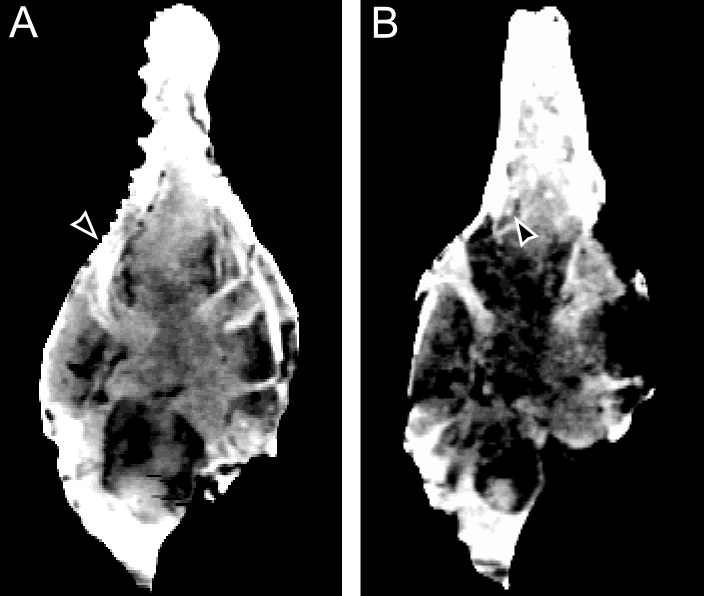


**Supplementary Figure 2. CT data for the skull of *Teyujagua paradoxa* (UNIPAMPA 653).** A, posterior extension of the maxillary tooth row (arrow); B, posterior extension of the dentary tooth row (arrow).

**
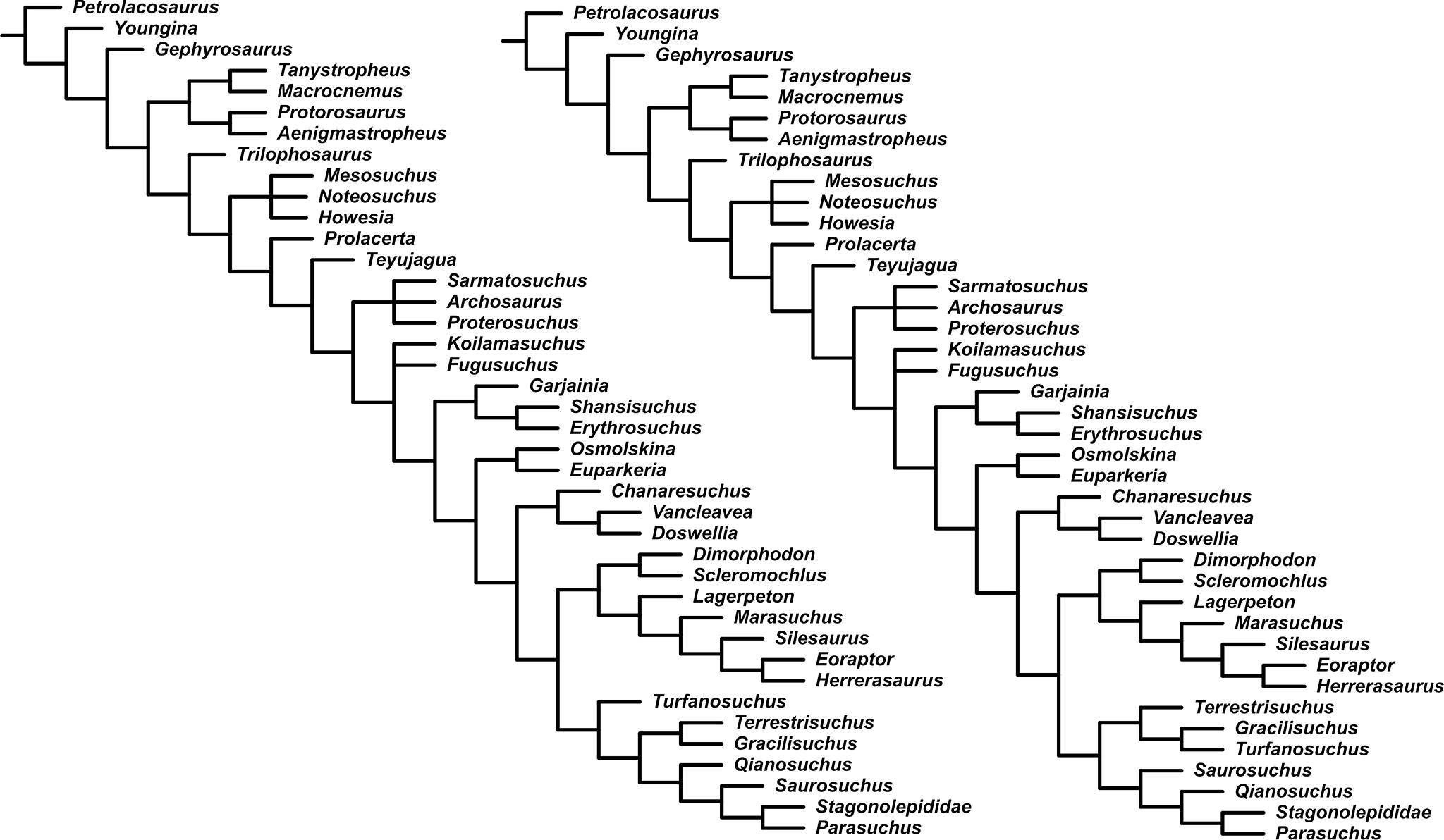
**

**Supplementary Figure 3. The two most parsimonious trees (872 steps) obtained in the cladistic analysis.**

**
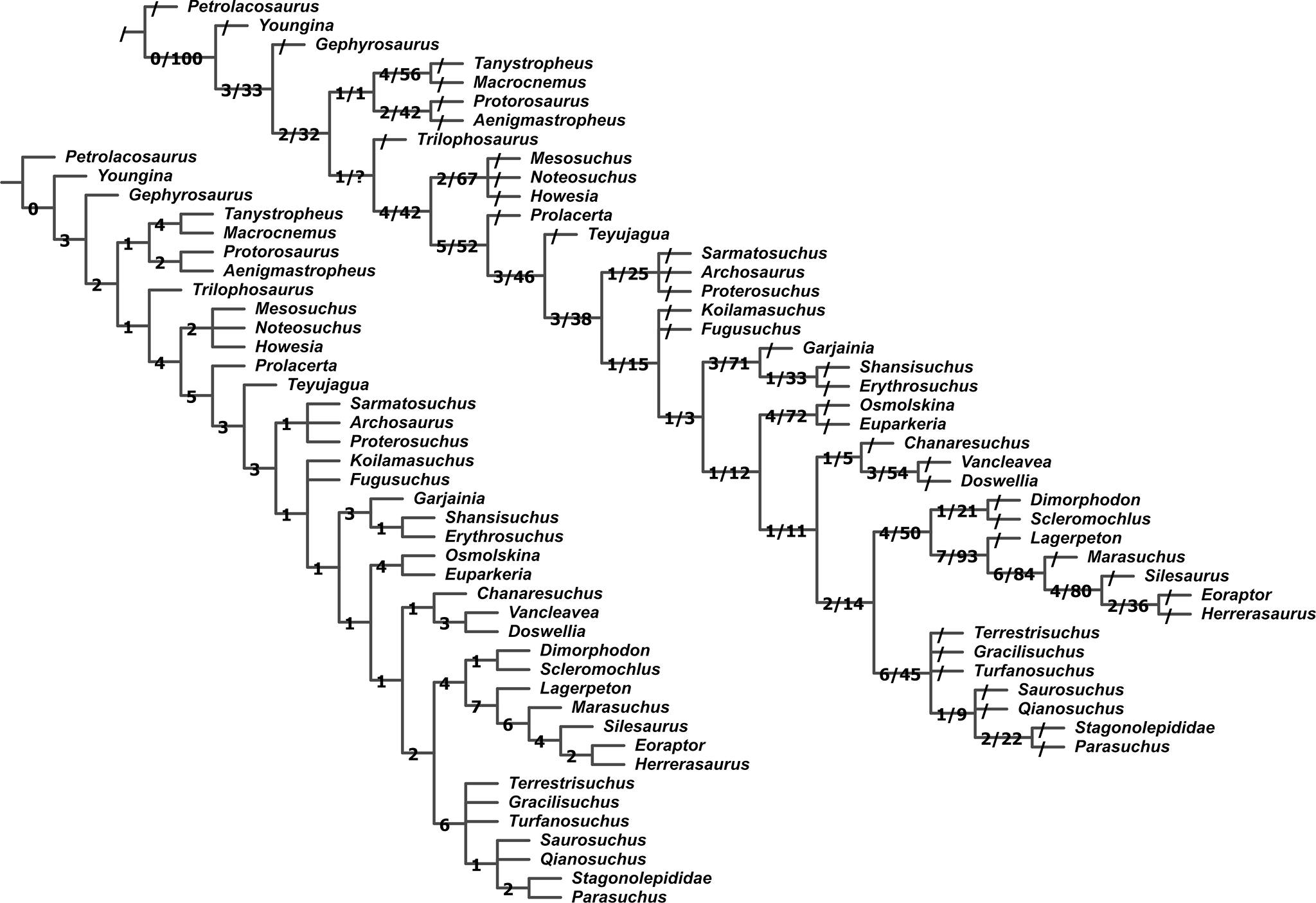
**

**Supplementary Figure 4. Bremer (lower left) and Bootstrap (upper right) support values for the strict consensus tree obtained by the cladistic analysis.**

**
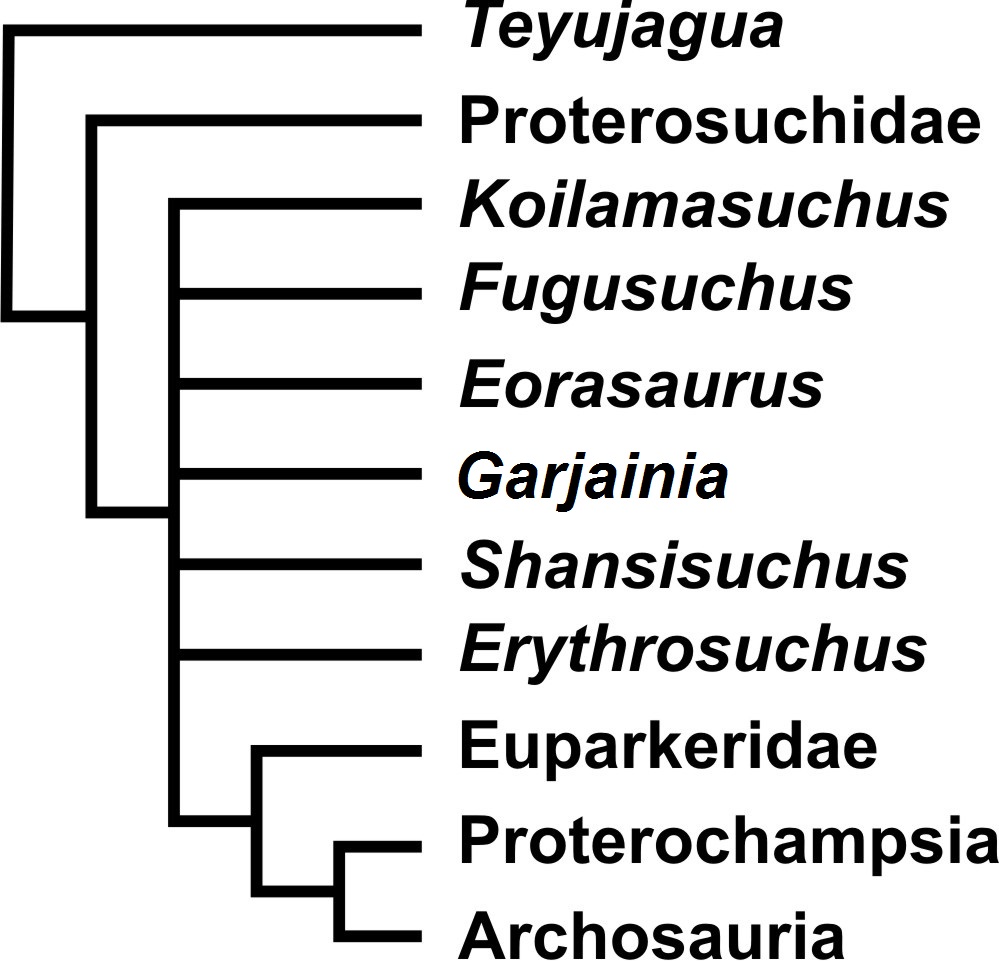
**

**Supplementary Figure 5. Strict consensus tree based on 14 most parsimonious trees for the analysis including *Eorasaurus* (for other taxa the topology is consistent with the strict consensus presented in Figure 4).**

**Supplementary Methods**

**Taxa and character selection.** The operational taxonomic units (OTUs) used in the analysis were selected as follows:

(i) Inclusion of all 28 OTUs from Ezcurra *et al*.^1^: *Chanaresuchus*, *Dimorphodon*, *Doswellia*, *Eoraptor*, *Erythrosuchus*, *Euparkeria*, *Fugusuchus*, *Garjainia triplicostata* (=“*Vjushkovia triplocostata*”), *Gracilisuchus*, *Herrerasaurus*, *Koilamasuchus*, *Lagerpeton*, *Marasuchus*, *Mesosuchus*, *Osmolskina*, *Parasuchus*, *Prolacerta*, *Proterosuchus*, *Qianosuchus*, *Sarmatosuchus*, *Saurosuchus*, *Shansisuchus*, *Silesaurus*, *Scleromochlus*, *Stagonolepis*, *Terrestrisuchus*, *Turfanosuchus*, *Vancleavea*;

(ii) inclusion of 16 additional OTUs from Ezcurra *et al*.^2^: the basal diapsid *Petrolacosaurus* as an outgroup; the basal neodiapsid *Youngina*; the basal rhynchochephalian *Gephyrosaurus* representing Lepidosauromorpha; and almost all Archosauromorpha used in their analysis (the only exception being *Eorasaurus*, which was scored separately), namely, *Aenigmastropheus*, *Protorosaurus*, *Macrocnemus*, *Tanystropheus*, *Trilophosaurus*, *Howesia*, *Noteosuchus*, *Archosaurus, Mesosuchus**, *Prolacerta**, *Euparkeria**, *Proterosuchus**, and *Erythrosuchus** (*indicates congruent OTUs with Ezcurra *et al*.^1^.

These taxa and the new taxon described in this paper, *Teyujagua*, totalled 40 OTUs. The character list was derived from Ezcurra *et al.*^1,2^.Characters were selected for inclusion in the present analysis as follows:

(i) Only morphological characters with character scores that are variable within the selected OTUs were included (i.e. if the same state is scored in the literature for either all or only one selected OTU –independently of whether missing data is present or absent – the character was not included in the present analysis);

(ii) Congruent characters present in two previous analyses were reconsidered, choosing the one with the original description and states, or with modification to the description and/or states;

(iii) Two completely new morphological characters were included.

This resulted in a total of 252 morphological characters.

**List of characters used in this study.** As described above, the characters used in the current phylogenetic analysis are derived primarily from the character-taxon matrices published by Ezcurra *et al*.^1, 2^. These studies themselves represent revised and expanded versions of previously published data sets (see the paper cited above for information on the original formulation of characters).

1. Teeth, tooth attachment: subthecodont (= protothecodont) (0); ankylothecodont (1); pleurodont (2); acrodont (3); thecodont (4).

2. Teeth, distal curvature of marginal teeth: present (0); absent (1).

3. Teeth, magnitude of distal curvature of marginal teeth: slight (0); strong (1).

4. Teeth, serrations on crown: absent (0); present (1).

5. Teeth, lateral compression of marginal dentition: only distally or nowhere (0); over two-thirds of tooth (1).

6. Vomerine teeth: present (0); absent (1).

7. Palatine, palatal teeth: present (0); absent (1).

8. Pterygoid, teeth on transverse flange: single row on edge (0); additional teeth anterior to single row (or no rows recognizable) (1); absent (2).

9. Pterygoid, teeth on palatine ramus: present in two fields (0), present in one field (1), present in three fields (2); absent (3).

10. Skull length: less than (0); more than (1) 50% of length of the presacral vertebral column.

11. External naris, position: marginal (minimal distance between nares ≥ 0.35 snout width at same level) (0); close to midline (1); confluent (2).

12. Antorbital fenestra: absent (0); present (1).

13. Maxilla, antorbital fossa exposed in lateral view: absent (0); present (1).

14. Dorsal margin of antorbital fossa is a shelf/ridge that extends across lacrimal, prefrontal, frontal portion of orbital rim, and postorbital: absent (0); present (1).

15. Supratemporal fenestra (= dorsal temporal fenestra): absent (0); present, postfrontal does not enter (1); present, postfrontal enters (2).

16. Infratemporal fenestra (= lateral temporal fenestra): absent (0); present, quadratojugal excluded (1); present, quadratojugal enters or is absent (2).

17. Shape of infratemporal fenestra: elliptical or subrectangular (0); trapezoidal, with dorsal margin much shorter than ventral margin (1).

18. Parietal, size of pineal foramen: large, more than 25% of mid-parietal length (0); small, less than 25% of mid-parietal length (1); absent (2).

19. Postorbital region, ventral temporal bar (zygomatic arch): absent (no fenestra) (0); tall, occupying more than 20% of skull height (1); narrow but complete, occupying less than 20% of postorbital skull height (2); incomplete (3); absent (with fenestra) (4).

20. Premaxilla, number of teeth: ≥ 5 (0); 2 to 4 (1); 0 (2).

21. Premaxilla, downturned alveolar margin: no (0); slightly (1); strongly (2).

22. Premaxilla, contact with prefrontal: absent (0); present (1).

23. Maxilla, tooth number: 25 or fewer (0); 26 or more (1).

24. Maxilla, number of tooth rows: single row (0); multiple rows (1).

25. Palatal processes on anteromedial surfaces of the maxillae: absent (0); present (1).

26. Maxilla, dorsal process: absent (0); starts just behind external naris, extends to level of dorsal narial margin (1); spike-like, just in front of orbit, overlies lacrimal (2); massive, pillar-like, extends above narial margin (3).

27. Maxilla, contact with prefrontal: absent (0); present (1).

28. Maxilla, caniniform region: region absent (0); region present (1); one or two caniniform teeth present (2).

29. Maxilla, anterior maxillary foramen: absent (0); present (1).

30. Maxilla, ventral margin: straight, concave or sigmoid (0); convex (1).

31. Maxilla, posterior extension: at level or posterior to posterior orbital border (0); anterior to posterior orbital border (1).

32. Maxilla, orbital exposure: absent (0); present (1).

33. Septomaxilla, shape: pillar-like (0); curled sheet (1); absent (2).

34. Ratio of lengths of nasal and frontal: equal or less than 1(0); more than 1(1).

35. Location of nasolacrimal canal foramen/foramina: in lacrimal (0); between lacrimal and prefrontal (1).

36. Exposure of the lacrimal on the skull roof in dorsal view: absent (0); present (1).

37. Lacrimal, length: participates in margin of external naris (0); does not reach external naris (1).

38. Prefrontal, suture with nasal: parasagittal, at least in its caudal third (0); anterolateral (1).

39. Frontal, suture with nasal: interdigited suture, almost 90º with sagittal axis (0); medial half more anterior to lateral half, with anterior projection of frontal on midline (1); medial half more anterior to lateral half, with posterior excavation of frontal on midline (2).

40. Frontal, shape of dorsal surface next to sutures with postfrontal and parietal: flat to slightly concave (0); longitudinal depression with deep pits is present (1).

41. Frontal, orbital border: absent or narrow (less than 1/3 of total length of orbit) (0); broad and forms most of dorsal edge (equal or more than 1/3 of total length of orbit) (1).

42. Frontal, posterolateral process: absent, fr-par suture forming right angle to parasagittal plane (0); absent or very short, fr-par suture forming obtuse angle to parasagittal plane (1); long, narrow, fr-par suture forming acute angle with parasagittal plane (2).

43. Postfrontal: equivalent in size to the postorbital (0); reduced to less than half the dimensions of the postorbital (1); absent (2).

44. Postfrontal, shape of dorsal surface: flat or slightly concave towards raised orbital rim (0); depression present with deep pits (1).

45. Depression on descending process of postorbital: absent (0); present (1).

46. Postorbital, posterior process if temporal fenestrae are absent reaches supratemporal or if at least one fenestra is present extends up to or beyond posterior margin of fenestrae: absent (0); present (1).

47. Parietal, extension over interorbital region: absent or marginal (0); present (1).

48. Parietal, sagittal crest: absent (0); present (1).

49. Parietal, median contact between both parietals: suture: present (0); fused with loss of suture (1).

50. Skull roof, distinct posterior emargination in late ontogeny: absent (0); present (1).

51. Squamosal, posterodorsal process: absent (0); present (1).

52. Posterior end of the squamosal: does not extend posterior to the head of the quadrate (0); extends posterior to the head of the quadrate (1).

53. Posterior process of the squamosal: straight (0); ventrally curved (1).

54. Squamosal overhanging quadrate and quadratojugal laterally: absent (0); present (1).

55. Squamosal, ventral process: broad, with proximal length approximately equal to dorsoventral height (0); narrow, with proximal length less than dorsoventral height (1); squamosal confined dorsally (2).

56. Projection of the ventral process of the squamosal: posteroventrally directed, vertical, or less than 30◦ from the vertical (0); anteroventrally directed at 30◦ or more (1).

57. Anterior end of jugal: enters into antorbital fenestrae (0); excluded by the contact of the maxilla and lacrimal (1).

58. Base of the posterior process of the jugal in lateral view: tapering slightly (0); semi-elliptical, with a ventral expansion (1).

59. Length of the posterior process of jugal: greater than (0); less than (1) half of total jugal length.

60. Anterior process of jugal: slender and tapering (0); broad and expanded anteriorly (1).

61. Quadratojugal, anterior process: present (0); absent (1).

62. Quadratojugal, anterior extent: maxilla-quadratojugal suture (0); extending anterior to ventral portion of squamosal, but not contacting maxilla (1); ≤ anterior extent of ventral portion of squamosal (2); quadratojugal absent (3).

63. Quadrate dorsal head in lateral aspect: hidden by squamosal (0); exposed (1).

64. Supratemporal: broad element of skull table (0); slender, in parietal and squamosal trough (1); absent (2).

65. Postparietal, size: sheet-like, both together not much smaller than suproccipital in state when the posttemporal fenestra is small (because of broad, plate-like dorsal process of suproccipital) (0); small, splint-like (1); absent (2).

66. Pterygoids, contact between each other: join cranially (0); remain separate (1).

67. Pterygoid, palatal process: extends anterior to the anterior limit of the palatine (0); forms oblique suture with palatine but process ends before reaching anterior limit of palatine (1); forms transverse suture with palatine (2).

68. Ectopterygoid: simple overlap of ectopterygoid and pterygoid (0); complex overlap between ectopterygoid and pterygoid (1); ectopterygoid absent (2).

69. Ectopterygoid, shape along suture with pterygoid: transversely broad (0); posteroventrally elongate and does not reach lateral corner of transverse flange (1); posteroventrally elongate and reaches corner of transverse flange (2).

70. Ectopterygoid, contact with maxilla: absent (0); present (1).

71. Ectopterigoid, posterior expansion in contact with jugal: absent (0); present (1).

72. Supraoccipital: excluded from dorsal border of foramen magnum by dorsomedial contact of exoccipitals (0); contributes to border of foramen magnum (1).

73. Association between paroccipital process and parietal: no contact (0); contact present immediately lateral to supraoccipital (1).

74. Posttemporal fenestra, size: large compared to suproccipital (narrow dorsal process of suproccipital tapers dorsally) (0); small (because of broad, plate-like dorsal process of suproccipital) (1); more or less foramen (2); absent (3).

75. Occipital neck, connecting the occipital condyle and the basioccipital body: present (0); absent (1).

76. Occipital condyle, position: even with craniomandibular joint (0); anterior to craniomandibular joint (1); posterior to craniomandibular joint (2).

77. Exoccipitals and opisthotics: discrete (0); fused (1).

78. Medial margin of exoccipitals: no contact (0); contact to exclude basioccipital from floor of braincase (1).

79. Opisthotic, paroccipital processes shape: slender (0); robust, with anteroposterior dimension at least one third greater than dorsoventral dimension (1).

80. Opisthotic, club-shaped ventral ramus: absent (0); present (1).

81. Opisthotic, paroccipital process attachment: ends freely (0); weak contact (1); strong contact (2).

82. Ventral ramus of the opisthotic: prominent (0); recessed (1).

83. ‘Pseudolagenar recess’ between ventral surface of the ventral ramus of the opisthotic and the basal tubera: present (0); absent (1).

84. Position on basisphenoid of foramina of cerebral branches of internal carotid arteries leading to the pituitary fossa: posterior/posteroventral (0); lateral (1).

85. Prootic, lateral surface: continuous and slightly convex (0); crista prootica present (1).

86. Prootic, contact with parietal: absent (0); present (1).

87. Prootic midline contact on endocranial cavity floor: absent (0); present (1).

88. Position of external abducens foramen on prootic: ventral surface (0); anterior surface (1).

89. Number of foramina for hypoglossal nerve: two (0); one (1).

90. Anteroventral process of prootic below trigeminal foramen: lateral ridge present (0); lateral ridge absent (1).

91. Stapes, shape: robust, with thick shaft (0); slender, rod-like shaft (1).

92. Stapedial foramen: present (0); absent (1).

93. Semilunar depression on parabasisphenoid: present (0); absent (1).

94. Parabasisphenoid plate between cristae ventrolaterales: intertuberal plate present (0); absent (1).

95. Parasphenoid cultriform process: simple (0); dorsoventrally constricted towards the base (1).

96. Base of cultriform process of parabasisphenoid: relatively short dorsoventrally (0); tall, with the dorsal edge extending up between clinoid processes and ventral parts of cristae prootica (1).

97. Orientation of basipterygoid processes: anterolateral (0); lateral (1).

98. Orientation of basisphenoid: horizontal (0); more vertical (1).

99. Basisphenoid midline exposure on endocranial cavity floor: present (0); absent (1).

100. Laterosphenoid: absent (0); present (1).

101. Laterosphenoid anterodorsal channel: absent (0); present (1).

102. Jaw occlusion: single-sided overlap (0); flat occlusion (1); blade and groove (2).

103. Lower jaw, distinct dorsal process behind the alveolar margin: absent, with a slightly convex dorsal margin behind the alveolar portion (0); present, formed by a dorsally well-developed surangular (1); present, formed by a dorsally well-developed coronoid and sometimes the posterodorsal ramus of the dentary (2); present, formed only by a dorsally hypertrophied coronoid bone (3).

104. Posterior extent of mandibular and maxillary tooth rows: subequal (0); unequal with the maxillary tooth extending further posteriorly (1).

105. External mandibular fenestra: absent (0); present (1).

106. External mandibular fenestra, position: at level of most posterior teeth of maxilla (0); posterior to most posterior teeth of maxilla (1). New character.

107. Dentary, number of tooth rows: one (0); two (1); more than two (2).

108. Posteroventral portion of the dentary: touch contact with surangular (0); laterally overlaps the anteroventral portion of the surangular (1).

109. Surangular, size of lateral exposure in relation to angular: surangular sub equal or less than angular (0); surangular widethan angular (1).

110. Surangular, lateral shelf: absent (0); present (1).

111. Surangular, anterior surangular foramen: absent (0); present (1).

112. Surangular, posterior surangular foramen: absent (0); present (1).

113. Retroarticular process, size: absent (0); small (1); large (2).

114. Retroarticular process: not upturned (0); upturned (1).

115. Cervico-dorsal vertebrae, anterior articular surface is situated higher than the posterior one: absent (0); present (1).

116. Centrum shape in presacrals 6–9 (or 10), in lateral view: sub-rectangular (0); parallelogram-shaped (1).

117. Cervical vertebrae, postaxial cervical intercentra: present (0); absent (1).

118. Cervical vertebrae, centra length: no longer than posterior dorsals (0); longer than posterior dorsals but fourth and fifth cervical centra less than three times their height (1); longer than posterior dorsals and fourth and fifth cervical centra equal or more than three times their height (2).

119. Cervical vertebrae, dimensions of postaxial anterior cervical neural spine: tall and long, with height and length approximately equal (0); long and low, with height lower than length (1); tall and narrow, with height higher than length (2).

120. Distal ends of cervical neural spines: no expansion (0); expansion present in form of a flat table (1).

121. Cervical vertebrae, postaxial cervical neural spines with an anterior overhang: absent (0); present (1).

122. Cervical, anterior dorsal, and mid-dorsal ribs, proximal tubercle that bears the articular facet for articulation with the vertebrae: poorly developed (0); long and distinct (1).

123. Cervical vertebrae, accessory process on anterolateral surface of anterior cervical ribs: absent (0); present (1).

124. Cervical ribs, slender and tapering at low angle to vertebrae: absent (0); present (1).

125. Cervical vertebrae, proximal rib heads: some or all holocephalous (0); all dichocephalous (1).

126. Dorsal vertebrae, intercentra: present (0); absent (1).

127. Dorsal vertebrae, mid-ventral surface of dorsal centra: rounded (0); ridged (with slightly swollen sides) (1); keeled (sharp edge) (2).

128. Centrum of dorsal vertebrae with a lateral fossa below the neurocentral suture: absent (0); present (1).

129. Dorsal vertebrae, ratio between transverse width of diapophysis and length of the centrum in anterior dorsal vertebrae: <0.65 (0); >0.75 (1).

130. Proportions of mid- and posterior dorsal centra: almost as long as tall (0); quite longer than tall (1).

131. Dorsal vertebrae, transverse processes in trunk: short (0); moderately long (1).

132. Dorsal vertebrae, zygapophyses close to each other medially, respectively, in anterior-middle dorsals: absent, zygapophyses laterally divergent beyond the lateral margin of the centrum (0); present, zygapophyses mainly oriented in the parasagittal axis (1).

133. Hyposphene-hypantrum accessory intervertebral articulations in trunk vertebrae: absent (0); present (1).

134. Orientation of mid-dorsal prezygapophyses: upwards (0); almost horizontal (1).

135. Dorsal vertebrae, anterior centrodiapophyseal lamina or paradiapophyseal lamina: absent (0); present (1).

136. Dorsal vertebrae, posterior centrodiapophyseal lamina: absent (0); present (1).

137. Dorsal vertebrae, prezygodiapophyseal lamina in posterior cervicals and anterior-middle dorsals: absent (0); present (1).

138. Dorsal vertebrae, postzygodiapophyseal lamina in anterior dorsals: absent (0); present (1).

139. Dorsal vertebrae, ratio of height of mid-dorsal neural spines from base of zygapophysis: maximum centrum height: ≤ 1.5 (0); > 1.5 (1).

140. Neural arches of mid-dorsals: deep excavation (0); no excavation or shallow excavation (1).

141. Dorsal vertebrae, dorsally opened pit lateral to the base of the neural spine: absent (0); present (1).

142. Distal ends of dorsal neural spines: no expansion (0); expansion present in form of a flat table (1).

143. Position of mid-dorsal neural spines: situated at midlength between the zygapophyses (0); posteriorly displaced from mid-length between the zygapophyses (1).

144. Sacral ribs, form and articulation of first rib with ilium: plate-like, contacts ilium in straight parasagittal articulation (0); distal end slightly dorsally expanded relative to shaft (1); entire rib dorsoventrally expanded and contacts ilium in C-shaped articulation (2).

145. Sacral ribs, second sacral rib: not bifurcate (0); bifurcate with posterior process pointed bluntly (1); bifurcate with posterior process truncated sharply (2).

146. Sacral and/or anterior caudal vertebrae, transverse processes and ribs: sutured to the vertebra (0); fused to the vertebra (1).

147. Caudal vertebrae, ratio of lengths of transverse processes and centra in anterior caudal vertebrae: equal or <1.0 (0); >1.0 (1).

148. Caudal vertebrae, anterior caudal neural spine height: moderately tall with height/length between >1.0 and <2.0 (0); low with height/length <1.0 (1); tall with height/length >2.0 and <3.0 (2); very tall with height/length >3.0 (3).

149. Chevrons, distal width of haemal spine: equivalent to proximal width (0); tapering (1); wider than proximal width (2).

150. Gastralia: present (0); absent (1).

151. Interclavicle, anterior half shape: +-shaped (anterior process present) (0); T-shaped (anterior process absent) (1).

152. Interclavicle, webbed between lateral and posterior processes: yes (head triangular or diamond-shaped) (0); no (rather sharp angles between processes) (1).

153. Interclavicle, anterior margin with a median notch: absent (0); present (1).

154. Interclavicle, posterior stem: little change in width along entire length (0); expansion present (1).

155. Scapula length: less than (0) or more than (1) twice the maximum anteroposterior width; more than three times the maximum anteroposterior width (2).

156. Scapula, anterior margin: straight, at least dorsally (0); convex along entire length (1).

157. Scapulocoracoid notch at anterior junction of scapula and coracoid: absent (0); present (1).

158. Forelimb–hind limb length ratio: more than 0.55(0); less than 0.55 (1).

159. Radius-humerus, length ratio: < 0.68 (0); 0.68 to 0.82 (1); > 0.82 (2).

160. Humerus, ratio of width of distal head to shaft length: ≥ 0.3 (0); < 0.3 (1).

161. Deltopectoral crest on humerus: rounded (0); subrectangular (1).

162. Deltopectoral crest: elongate and apex situated at a point corresponding to less (0); or more than (1) 38% down the length of the humerus.

163. Humerus, entepicondyle: moderately large (0); strongly developed at maturity (1).

164. Humerus, entepicondylar foramen: present (0); absent (1).

165. Humerus, ectepicondylar region: foramen, process bridged (0); supinator process present, groove present (1); process, groove and foramen absent (2).

166. Humerus, torsion between proximal and distal ends: around 45° or more from one another (0); 20° or less from one another (1).

167. Humerus, capitellum (radial condyle) and trochlea (ulnar condyle): strongly developed as distinct ball-shaped structures (0); poorly developed butdistinct from the ectepicondyle and entepicondyle (1); absent (2).

168. Transverse width of the distal end of the humerus: less than (0); equal or more than (1) 2.5 times the minimum width of the shaft.

169. Ulna, olecranon process in lateral view: tapering toward its distal end (0); subrectangular or slightly expanded towards its distal end (1).

170. Ulna, olecranon process: absent or very low (0); prominent but lower than its transverse depth at base (1); strongly developed, being higher than itstransverse depth at base (2).

171. Carpus, lateral centrale carpi: large (0); small or absent (1).

172. Metacarpus, fourth metacarpal: longer than metacarpal III (0); equal or shorter than metacarpal III (1).

173. Manual digit IV: five (0); four (1); fewer than four (2) phalanges.

174. Acetabulum: imperforate, with a ventral acetabular wall projection anteriorly displaced from mid-length of the acetabulum (0); imperforate, with a ventral acetabular wall projection centered at mid-length of the acetabulum (1); perforated (2).

175. Acetabulum: mainly laterally oriented (0); mainly ventrally oriented (1).

176. Acetabular antitrochanter on ilium and ischium: absent (0); present (1).

177. Preacetabular process: absent (0); present but poorly developed (1); present and well developed (2).

178. Dorsal margin of ilium: convex with broadly rounded anterior and posterior ends (0); straight or with only a portion slightly convex and bluntly pointed anterior and posterior ends (1).

179. Ilium, iliac blade: posterior process only (0); large posterior process and smaller anterior process (1); equally developed anterior and posterior processes (2); large anterior projection (3).

180. Ilium, anteroposterior development of the iliac blade: well developed (0); reduced (1).

181. Maximum length of the iliac blade: less than (0); more than (1) 3 times its maximum height.

182. Brevis shelf on ventral surface of postacetabular part of ilium: absent (0); present (1).

183. Brevis fossa with sharp margins on the ventral surface of the postacetabular process of the ilium: absent (0); present (1).

184. Posterior border of the iliac ischiadic peduncle: vertical or poorly posteriorly expanded (0); strongly posteriorly expanded resulting in a tapering projection, with a posterior border settled at 45◦ or lower to the longitudinal axis of the ilium (1).

185. Pubic tubercle: prominent (0); reduced to rugosity (1).

186. Dorsal margin of the pubic peduncle forming an angle lower than 45◦ to the longitudinal axis of the bone: absent (0); present (1).

187. Pubis, pectineal process: absent (0); present (1).

188. Pubis, anterior apron: absent (0); present (1).

189. Pubic length: shorter than ischium (0); longer than ischium (1).

190. Pubic length: less than (0); more than (1) twice the length of the acetabulum.

191. Pubic acetabular margin, posterior portion: continuous with anterior portion (0); recessed (1).

192. Pubic tuber in lateral aspect: anteroventrally directed (0); strongly downturned (1).

193. Pubis, form in lateral view: plate-like (0); rod-like and curved posteriorly (1); rod-like and straight (2).

194. Width of the conjoined pubes: less than (0); greater than 75% of their length (1).

195. Ischial length: less than (0) or more than (1) twice the anteroposterior length of the acetabulum.

196. Femur, maximum length/distal width ratio: < 4 (0); ≥ 4 (1).

197. Femoral-humerus, shaft diameters: femur = 150% humerus (0); more or less equal (up to 120%) (1).

198. Femoral head: not distinctly offset (0); distinctly offset (1).

199. Femoral head articular surface: limited extent (0); extends under head (1).

200. Fossa trochanterica on proximal face of femoral head: absent (0); present (1).

201. Trochanteric shelf: absent (0); present (1).

202. Femoral anterior trochanter: absent (0); present (1).

203. Fourth trochanter of femur: absent (0); present (1).

204. Intertrochanteric fossa on ventral aspect of proximal portion of femur: present (0); absent (1).

205. Femur, shaft: diameter constant or widening distally (0); diameter distally narrowed (1).

206. Femur, distal articular surface: uneven, fibular condyle projecting distinctly beyond tibial condyle (0); both condyles prominent and approximately at same level (1); both condyles do not project beyond shaft (distal articular surface concave or almost flat) (2).

207. Posterior proximal tubercle on femur: well developed (0); indistinct to absent (1).

208. Tibia-femur ratio: less than 1(0); equal to or more than 1 (1).

209. Cnemial crest on tibia: absent (0); present (1).

210. Tibia with posterolateral flange, with receiving depression on dorsal aspect of astragalus: (0) absent; (1) present.

211. Fibula and calcaneum shape: unreduced (0); fibula tapering and calcaneum reduced in size (1).

212. Fibular anterior trochanter (insertion site for iliofibularis muscle): low rugosity (0); robust pendent trochanter (1).

213. Proximal tarsals, astragalus-calcaneum articulation: flat (0); concave-convex (1); foramen on calcaneum, articulation expanded (2); sutured or fused (3).

214. Proximal tarsus, foramen for the passage of the perforating artery between the astragalus and calcaneum (= perforating foramen): present (0); absent (1).

215. Astragalar tibial facet: concave (0); saddle-shaped (1).

216. Ventral astragalocalcaneal articular facet: small (0); larger than dorsal articulation (1).

217. Crural facets on astragalus: separated by a nonarticular surface (0); continuous (1).

218. Astragalar anterior ascending process: absent (0); present, occupying most of the anteroposterior depth of the astragalus (1); present, restricted to the anterior half of the astragalar depth (2).

219. Astragalar posterior (= ventral) groove: present (0); absent (1).

220. Astragalar anteromedial corner shape: obtuse (0); acute (1).

221. The dorsolateral margin of the astragalus: overlaps the anterior and posterior portions of the calcaneum equally (0); the posterior corner of the dorsolateral margin of the astragalus dorsally overlaps the calcaneum much more than the anterior portion (1).

222. Calcaneal tuber: prominent (0); rudimentary or absent (1).

223. Calcaneal tuber shaft proportions: taller than broad (0); broader than tall (1).

224. Orientation of calcaneal tuber: lateral (0); deflected more than 45◦ posterolaterally (1).

225. Calcaneal tuber distal end: anteroposteriorly compressed (0); rounded (1).

226. Calcaneal tuber distal end, with vertical median depression: absent (0); present (1).

227. Articular surfaces for fibula and distal tarsal IV on calcaneum: separated by a non-articular surface (0); continuous (1).

228. Hemicylindrical calcaneal condyle for articulation with fibula: absent (0); present (1).

229. Calcaneal proximal articular face: convex or flat (0); concave (1).

230. Calcaneal distal articular face: transverse width greater (0); or less (1) than 35% of that of the astragalus.

231. Distal tarsus, number of pedal centralia: both medial and lateral centralia present (0); only lateral pedal centrale and does not contact tibia (1);only lateral pedal centrale and contacts the tibia (2); pedal centralia absent as sperate ossifications (3).

232. First and second distal tarsals: present (0); absent (1).

233. Distal tarsal 4: transverse width broader than (0); subequal to (1) distal tarsal 3.

234. Distal tarsal 4, size of articular facet for metatarsal V: more than (0); less than (1) half of lateral surface of distal tarsal 4.

235. Metatarsus configuration: metatarsals diverging from ankle (0); compact metatarsus with metatarsals I–IV tightly bunched (1).

236. Metatarsal midshaft diameters: I and V subequal or greater than II–IV (0); I and V less than II–IV (1).

237. Metatarsal I length relative to length of metatarsal III: 50–75% (0); 75% or greater (1).

238. Metatarsus, ratio of lengths of metatarsals I and IV: equal or >0.42 (0); <0.42 and equal to or >0.32 (1); equal to or <0.32 (2).

239. Metatarsal II midshaft diameter: less than or equal to (0); more than (1) the midshaft diameter of metatarsal I.

240. Metatarsal II–IV length: less than (0); equal or greater than (1) 23% of the length of the femur plus the tibia.

241. Metatarsal IV: nearly the same midshaft diameter as metatarsal III (0); reduced where the midshaft diameter is less than metatarsal III (1).

242. Metatarsal IV: longer than metatarsal III (0); about the same length or shorter than metatarsal III (1).

243. Metatarsus, fifth metatarsal: straight (0); hooked (1).

244. Metatarsus, fifth distal tarsal: present (0); absent (1).

245. Ratio of lengths of pedal digits V and I: more than 1(0); less than 1 (1).

246. Ratio of lengths of pedal digits III and IV: equal or less than 1(0); more than 1 (SA) (1).

247. Phalanges/phalanx on pedal digit V: present (0); absent (1).

248. Dorsal body osteoderms: absent (0); present in one or more rows (1).

249. Dermal osteoderms on ventral side of body: absent (0); articulate and form a carapace (1).

250. Osteoderm sculpture: absent (0); present (1).

251. Premaxilla, lenght of body parallel to dental margin: lenght less than 3 times the heigth (0); lenght equal or more than 3 times the height (1). New character.

252. Premaxilla, posteroventral process extension: at level or anterior to posterior margin of external naris (0); posterior to external naris (1). New character.

**Scoring OTUs.** The OTUs were scored based on specimens described in the literature and on previous first-hand examination of taxa by F.L.P., M.A.G.F. and M.B.L. When first-hand observation of specimens was not possible, character states described in the original description/re-description of taxa were thoroughly checked through a critical analysis of the pertinent literature (including text and figures). The data matrix was elaborated using the Mesquite software version 3.03^3^.

**Character-taxon matrix.**

*Petrolacosaurus*

00000?010?00??1201100000?002101010?000101200?010000???00??1001010000000101?0??0?0??00?????00???????0?0000?0?00000??001110?10001?010?0?10001?1???0000110010000?2100001001?200?0??00100??00?1000000??0000000000000000000????????????????10???01000000000000?00

*Youngina*

00000?002?00??2201210010?110001010?0111012?00110000???10???0011100100?01?010?0011?0100????000????0?0?0010?0??000100000000000100?0?000010000?00??0?1121110000001000101011-0000000000100?00010000000111000000001000000000000??0100??000010?????1????00???00?00

*Gephyrosaurus*

21?00?022?10??2201300010?1101011????11101100?000100???01??1012122010110????1???????0???????????????0?0210?0?1010200000?00?00101?0?00?00000??0????11?20110??????0001000?1?(1 2)???0??00010??00?10??????1??00000000?000000210??0???1????000?3???????????11??????00

*Aenigmastropheus*

??????????????????????????????????????????????????????????????????????????????????????????????????????????????????10???0??????1????1?01110??0?????????????????????111?0?02??????????????????????????????????????????????????????????????????????????????????

*Protorosaurus*

(0 2)(0 1)000???2?10??1??(1 2)310010?10010102???1??011?0?001(0 1)10???1????????2?????????0?0??001????????????????????00?0?0?10?0211012111?11(0 1)1??0?11??11111?1???0002201010000020??1111(0 1)?0200????????????0?01???????1?0??????11?1????00???????1????????10????00????1?00000?00

*Macrocnemus*

00100???0??0??1?02300000?1101010?????1101200?011010????0??10021????????????????????????????????????0?0?00?0?10?0?01002101?11112?0?00??1?100?0???211?20001000?02100011?2?-0?(0 1)?(0 2)????10????0?010??????100000000110100000000?0??0100??00?02000000(0 1)00001110000?11

*Tanystropheus*

0(0 1)(0 1)00?02(1 2)?10??22?(0 1)300000?1100010????11?01(0 1)?0?011110???1??????3??2000??0??0?0??0?0???1?????1????????0?00?0?0?1000201012101?11(0 1)12?0?10??11110?0???011020???0?0?0(0 1)10001112?-011?0???010????0?01???????100??????11?1????00???????1???????????????0????11???00???

*Trilophosaurus*

11?00?123?00??20?2020000?1?01001???011100110?001010???2???1???0221???10100?1?0001??01?????11?????0?0?0200?0?10002(0 1)110(0 1)001011101?1110?000111?0???010(0 2)0?10010?002(0 1)00111101011000000010?0?10010000000111000000001?000001000?0???000??00001000000100001100000?01

*Howesia*

11?00?02??2???2??23??1-1?(1 2)1??11??0?010110101?001100???10??0012002?(0 1)11111?1?1???????0???????????????0?11?0?2?1?0021?01????????00???1????000??1???21122001??????????01?1???????0??0?100??10?110???????????????01??????10???????0????????20000???0?00?1????????

*Noteosuchus*

???????????????????????????????????????????????????????????????????????????????????????????????????????????????????????0?0???00??11???00001?????(1 2)112?0?????????????????????0?0????000??10??1???????0????????01??????10???????0????????(1 2)0000002000?11????????

*Mesosuchus*

400000022020??22013111?10110101020001111010100011000?010?0001200200111110111001110?01???0???01??00?0?1100?20100021?01120001110000110000000001010211(2 3)2000110000?000011?21-010?00000(0 1)0000100110000000010000000010000001000000000000000002000000200001100000?00

*Prolacerta*

101010000010??120(1 2)30100001001010110011200200001001100010?0001211210?00?1010100111000100000??00000000?0010?00101121100211101110(0 1)001110?101000101011102?001000002000011021?01000000010000000010000000110000000010000001000000000000000002000000000001100000?00

*Teyujagua*

40111?????20??2211311000?1100011?10011100000000001100?10-000??112????????????????????????????????????0??100011?121?????00??1??????????????????????????????????????????????????????????????????????????????????????????????????????????????????????????????00

*Proterosuchus*

10111000201100221(1 2)(2 3)020(0 1)001001100110011(0 1)010000100011000100(0 1)001211010?20101(1 3)110111200011?0?011001010?1000111000111211001210011100?1?1101100(0 1)001010211(0 2)2001100000??00011?2?00???00001101000000100000000?000000001000000100000000000000000200000000000110?000?11

*Archosaurus*

1?????????1???2????02?????????????????0010????0001???????????????????????????????????????????????????0????????????????????????????????????????????????????????????????????????????????????????????????????????????????????????????????????????????????????1?

*Sarmatosuchus*

10111?022?1????????02?????????????????101?????????100?10?100??1????????1??1?1111?0001???10??00?011???0????0???????10012000??0001111???10??????????????????001????????????0????????????????????????????????????????????????????????????????????0????????00?1?

*Fugusuchus*

?01?1?????110012122?100??100?000?1?1112?001?0100011000000100020?1??????011?11111200011?110??0010?0?1?0???????????????????????0????????????????????????????????????????????????????????????????????????????????????????????????????????????????????????????01

*Koilamasuchus*

?????????????????????????????????????????????????????????????????????????????????????????????????????????????????????????0??1?01?1???1????0??01?????10?????????00????1?1?????00?21??1??0?1?????????????????????????????????????????????????????????????1?0??

*Erythrosuchus*

40111?1230111011022010001100001021101020001001000100?1001101010210??2010110111012110111111?10001111110011101010121100020011100211011001110101011000100????1100100101112110???000211010011101000000101000001002000000110010000000000000310000??000111?1010001

*Garjainia*

?01????23011???20???1???1????????1??????????0????????000?101??0????????01??11?01?0001?1110??0000?11110??1?????????10?021?1?000???0??00????10?011??????1?011110??01?????1?????00021??1001?1??00000010???????002000000??????????000?00????????????????????????

*Shansisuchus*

40111?123011??12022010?01100?010?1?01?2?001?01000100?000?100010?1??????01101110??0??1??111??0?01?1?1100111010101211000???1??0??1?0110011??10?0110??100????111010010?11?110???00021?01001100100000000100?00?002000000110?00?0100000001?3???001000111?0001?001

*Euparkeria*

40111002001110121221100011000010211110201110011001110010001102021111201101011001100011?100?1011011?1?0011101111121100101011101010101?111100011100102101?001100210001212000?1?0001110100011010001001100000011020000001100100010100000003100000000011101010001

*Osmolskina*

40111?020?111?121221100001??10???????0200210011001100010?0110?0?1???2??1??1?100???001???001101??11?1?001110??11?2110010?0????1??0?01?111100011?0??0110????11???10?012120?0???000111010001101???100????????1?02??????11000?00?01???0?0?3????????????????10?1?

*Chanaresuchus*

4011110211111102122010001100001021101?0?122?1000011100000001010221??20110101100?10001???11??10??10?1?001110101002010110001?0010101010011110110100?0010????1110210001212100???10021101000110100010001000000110200000011001000?0000000003100000110110111110010

*Doswellia*

4???0??21?????00?22??????????????????????02??000011110?0?00001022???2011010010?1?0?0????11??11??00???00?0?001?00?01011?100?101000111011???01?1000??1100001???????????????????10020110000010100010001?0000?01020????????????????????????????????????????101??

*Vancleavea*

40011????010??020220000011120001?1????20122?000001111001?10001022???????0?00?00???0??1???1???1???0?1?02???0110??201011?0????0101???100???1?1100??????0????01?0100001212100???00001110000?0?1??0???01100000010210000?110000000000?00000310000?0000?0????11001

*Turfanosuchus*

40111?121111111?12200000?1?0001021?11?2012011000011101111011020??????0????0?1????0?0???101??11??10???001110111002110?1?111?????101??????11?1?1?00?0???????11?0?100012121????2000211?10001101?10110?1?00000110200??0011011000?011111100????000?00100?00010001

*Qianosuchus*

40111?123011?012122000001100001021?01??01?1010100111101110110112??0?20?????????????0?????????1???1?1?001110111??211011100??0?1??011??111111?101???02201???011?????????????????0021101000110101111011?00?001?020000011?1110???0111?11?031??000000011????10000

*Parasuchus*

4011111231211012022000001100001001101??0110010000111111000010102201?2011010111011011??0101??10?11101000111011?002110112001?00100011101111?11?1100?022000011110000001112111?111002110100111010001001110000011020000011111100000101011003100000000011101011110

Stagonolepididae

41?1111230111012122000001100001021101??0121000100111(0 1)11100110(1 2)0220(0 1)?210?010210011011???001??110101?1?0011101110021101??0?1?0?10(0 1)?11?01????1??1110?00?01?011110100001112101?121102110100111011111201100000011020000011111100010111111003100000000011111011110

*Saurosuchus*

401111123011110?122100001100001021011??0121000000111101000110102200?20100111110110?1?10?11??110011010001?10?01??2?10102101?0?12000111011110111110?00?0?????1???????1?1???????010211010011101??112011?000001102000001111110000011111100310000000?011?1?010001

*Gracilisuchus*

40111??23011101212210000?100001021?11?201?111000011101111011020211???1??0??0?????01??????????1??10???001110011002110110011?0012??1??01????01?0110????0????11?01100012121?????0002110100011?111?12011?00000110200100011111000001111110031??00100001111?010001

*Terrestrisuchus*

401111123111100212200000?1000010?1?11?1?112?00000111012?10100?0221??0001001010??101?????????????11?1?001110101??2110110011?001?0011?00????01?0110?01?010001110210001212001?012012110100011011111101110000011020100001111100000111111003100001000011111010011

*Scleromochlus*

???????231111???1?200000?1?0?0??????1???1?0???1??????0??1????????1????????02????????????????????00???000110???00???011?????0?1?????????????????00?????????2001210001?120???1?100211010001???10?100?1100?0011020100001?0??0???00100???0311?101?01011??0100?1?

*Dimorphodon*

40101????1211??20?211?00????001??1????????1?0????????0??0010??0????????????2?????????????????????????000110?1?????101??0?1?001?1?1???1????01?010?????02???210021100111100??11(0 1)002110100?1???000100011100000?0201001011001010000?00?0003110101?000111?0100?10

*Lagerpeton*

?????????????????????????????????????????????????????????????????????????????????????????????????????????????????????????????1?0?1???1?????1?0110??2??2??????????????????????00021101000110100011011?100001102011010210?10110????010113?11110201000100100???

*Marasuchus*

?01?1??????1??????????00?1????????????????????????????????????????????????1?1??????1????01??11??00?1?0????????????111??00???01?1?1010(0 1)11110100110??02?????11?1211001?120?????10121101000110111011011?011111102011010210?1101?001001011311111001101010110????

*Silesaurus*

1??11??23011?????2?1000011?00010?1????201????????????????0?1?????0?????1??0?10???0?0???101??110000???0011?0?11012111111001?001?011011011110000110?02?02???10?0211001212010???001211011101101110111111001111102101110210?121101010010113???11??10010??1100?10

*Herrerasaurus*

401111123011100212210000?100001021111?10122?11000110000000110112201?20011101100110?1????0???110000?1?001100101012111111001?0011110011111110101120?0??02???10?1211101212011112201211011001101110121110111111102101110210?1201010100101131?1110011010111000?01

*Eoraptor*

40111?101011110202211000?100001021?11?10122?0000011100101011011220??201????2?????????????????????0?1?00111011100211111?0?1??011001011111110100120?02?02???1101111101212000?12201211011101101110121110111011102111110210?1211010?0010113101110011010111100?01

*Eorasaurus* (not included on the main analysis of this study):

??????????????????????????????????????????????????????????????????????????????????????????????????????????????????1?0[01]??????????1?11??1110??0???????????????????????????????????????????????????????????????????????????????????????????????????????????????

**Supplementary Results**

An additional cladistic analysis also was performed, excluding *Teyujagua*. This also recovered two most parsimonious trees, but with 864 steps, and an otherwise identical topology to those trees recovered by the analysis including *Teyujagua*. In this second analysis, Archosauriformes has 21 synapomorphies (Char. 4: 0 --> 1; Char. 12: 0 --> 1; Char. 17: 0 --> 1; Char. 42: 1 --> 0; Char. 46: 0 --> 1;Char. 58: 0 --> 1; Char. 65: 2 --> 1; Char. 69: 1 --> 2; Char. 73: 0 --> 1; Char. 78: 0 --> 1; Char. 81: 1 --> 2; Char. 86: 0 --> 1; Char. 89: 0 --> 1; Char. 97: 0 --> 1; Char. 100: 0 --> 1; Char. 105: 0 --> 1; Char. 110: 0 --> 1; Char. 128: 0 --> 1; Char. 178: 0 --> 1; Char. 181: 0 --> 1; Char. 252: 0 --> 1).

Comparing the two analyses, the synapomorphies of the Archosauriformes recovered in analysis 2 (excluding *Teyujagua*) are altered as follows when *Teyujagua* is included: five characters become synapomorphies of the clade *Teyujagua* + Archosauriformes (characters 4, 17, 42, 105, 110); five characters are maintained as synapomorphies of Archosauriformes (characters 12, 46, 58, 65, 252); and 11 characters are of ambiguous optimisation because their scores are not accessible for *Teyujagua* owing to problematic or absent skull material (characters 69, 73, 78, 81, 86, 89, 97, 100) or the relevant anatomical region is not preserved in the *Teyujagua* holotype (characters 128, 178, 181).

Another analysis was performed including *Eorasaurus.* Only 11 characters can be scored for this taxon (115, 117, 118, 129, 131, 132, 135, 136, 137, 138, and 141). The analysis resulted in 14 most parsimonious trees, with 873 steps. Most of the recovered topologies are similar to those recovered in the first phylogenetic analysis. The consensus tree differs by the presence of a polytomy including *Eorasaurus* in the clade composed of *Koilamasuchus*, *Fugusuchus* and erythrosuchid taxa and a clade composed of Euparkeriidae + Proterochampsia + Archosauria (Supplementary Fig. 5). This provides additional support for the archosauriform affinities of *Eorasaurus* and the existence of a ghost lineage of Archosauriformes starting in the middle Wuchiapingian, as discussed by Ezcurra *et al*.^2^.

**List of synapomorphies common to the two most parsimonious trees recovered in this study.**

*Petrolacosaurus*:

All trees: No autapomorphies;

Node *Youngina* + Archosauriformes:

All trees: No synapomorphies;

*Youngina*:

All trees: Char. 29: 1 --> 0; Char. 46: 0 --> 1; Char. 84: 0 --> 1; Char. 127: 1 --> 0; Char. 159: 2 --> 1; Char. 170: 1 --> 0;

Node *Gephyrosaurus* + Archosauriformes:

All trees: Char. 11: 0 --> 1; Char. 19: 2 --> 3; Char. 42: 2 --> 1; Char. 47: 1 --> 0; Char. 62: 1 --> 2; Char. 64: 1 --> 2; Char. 65: 0 --> 2; Char. 69: 0 --> 1; Char. 113: 1 --> 2; Char. 150: 1 --> 0; Char. 243: 0 --> 1; Char. 244: 0 --> 1;

*Gephyrosaurus*:

All trees: Char. 1: 0 --> 2; Char. 2: 0 --> 1; Char. 32: 0 --> 1; Char. 49: 0 --> 1; Char. 56: 0 --> 1; Char. 103: 01 --> 2; Char. 111: 0 --> 1; Char. 165: 1 --> 0; Char. 213: 0 --> 2; Char. 214: 0 --> 1; Char. 231: 12 --> 3;

Node Protorosauria + Archosauriformes:

All trees: Char. 48: 0 --> 1; Char. 50: 0 --> 1; Char. 115: 0 --> 1; Char. 124: 0 --> 1; Char. 131: 0 --> 1; Char. 137: 0 --> 1; Char. 164: 0 --> 1; Char. 166: 0 --> 1;

Node Protorosauria (Protorosauridae + Tanystropheidae):

All trees: Char. 118: 1 --> 2; Char. 126: 0 --> 1; Char. 136: 0 --> 1; Char. 187: 1 --> 0; Char. 205: 0 --> 1; Char. 208: 0 --> 1;

Node Protorosauridae (*Protorosaurus* + Aenigmastropheus):

All trees: Char. 132: 0 --> 1; Char. 170: 1 --> 2;

*Aenigmastropheus*:

All trees: No autapomorphies;

*Protorosaurus*:

All trees: Char. 120: 0 --> 1; Char. 138: 0 --> 1; Char. 141: 0 --> 1;

Node Tanystropheidae (Tanystropheus + Macrocnemus):

All trees: Char. 47: 0 --> 1; Char. 127: 1 --> 2; Char. 160: 0 --> 1; Char. 163: 1 --> 0; Char. 167: 01 --> 2; Char. 170: 1 --> 0;

*Macrocnemus*:

All trees: Char. 9: 2 --> 0; Char. 42: 1 --> 2; Char. 131: 1 --> 0; Char. 145: 0 --> 2;

*Tanystropheus*:

All trees:

Char. 29: 1 --> 0; Char. 49: 0 --> 1; Char. 62: 2 --> 3; Char. 138: 0 --> 1; Char. 159: 2 --> 01;

Node *Trilophosaurus* + Archosauriformes:

All trees: Char. 1: 0 --> 1; Char. 41: 1 --> 0; Char. 213: 0 --> 1; Char. 222: 1 --> 0;

*Trilophosaurus*:

All trees: Char. 2: 0 --> 1; Char. 7: 0 --> 1; Char. 9: 2 --> 3; Char. 11: 1 --> 0; Char. 16: 2 --> 0; Char. 19: 3 --> 0; Char. 20: 01 --> 2; Char. 31: 1 --> 0; Char. 32: 0 --> 1; Char. 43: 0 --> 1; Char. 55: 1 --> 2; Char. 103: 01 --> 2; Char. 116: 0 --> 1; Char. 129: 0 --> 1; Char. 138: 0 --> 1; Char. 147: 1 --> 0; Char. 149: 2 --> 0; Char. 252: 0 --> 1;

Node Rhynchosauria + Archosauriformes:

All trees: Char. 21: 0 --> 1; Char. 59: 1 --> 0; Char. 64: 2 --> 1; Char. 71: 0 --> 1; Char. 74: 0 --> 1; Char. 79: 0 --> 1; Char. 127: 1 --> 0; Char. 141: 0 --> 1; Char. 151: 1 --> 0; Char. 163: 1 --> 0; Char. 167: 01 --> 2; Char. 170: 1 --> 0; Char. 195: 1 --> 0;

Node Rhynchosauria:

All trees: Char. 11: 1 --> 2; Char. 22: 0 --> 1; Char. 24: 0 --> 1; Char. 40: 0 --> 1; Char. 44: 0 --> 1; Char. 49: 0 --> 1; Char. 50: 1 --> 0; Char. 64: 1 --> 0; Char. 102: 0 --> 1; Char. 107: 0 --> 2; Char. 117: 0 --> 1; Char. 137: 1 --> 0; Char. 148: 1 --> 2; Char. 238: 01 --> 2;

*Howesia*:

All trees: Char. 2: 0 --> 1; Char. 30: 0 --> 1; Char. 38: 1 --> 0; Char. 152: 0 --> 1;

*Noteosuchus*:

All trees: Char. 179: 1 --> 0;

*Mesosuchus*:

All trees: Char. 1: 1 --> 4;

Node *Prolacerta* + Archosauriformes:

All trees: Char. 3: 0 --> 1; Char. 5: 0 --> 1; Char. 34: 0 --> 1; Char. 48: 1 --> 0; Char. 51: 0 --> 1; Char. 70: 1 --> 0; Char. 104: 0 --> 1; Char. 112: 0 --> 1; Char. 132: 0 --> 1; Char. 187: 1 --> 0;

*Prolacerta*:

All trees: Char. 8: 2 --> 0; Char. 9: 2 --> 0; Char. 15: 2 --> 1; Char. 39: 1 --> 2; Char. 42: 1 --> 2; Char. 47: 0 --> 1; Char. 69: 1 --> 0; Char. 118: 1 --> 2; Char. 120: 0 --> 1; Char. 145: 02 --> 1; Char. 148: 1 --> 0; Char. 166: 1 --> 0;

Node *Teyujagua* + Archosauriformes:

All trees: Char. 4: 0 --> 1; Char. 17: 0 --> 1; Char. 42: 1 --> 0; Char. 105: 0 --> 1; Char. 110: 0 --> 1;

*Teyujagua*:

All trees: Char. 11: 1 --> 2; Char. 32: 0 --> 1;

Node Archosauriformes (*Proterosuchus* + Archosauria):

All trees: Char. 12: 0 --> 1; Char. 46: 0 --> 1; Char. 58: 0 --> 1; Char. 65: 2 --> 1; Char. 252: 0 --> 1;

Node Proterosuchidae (*Proterosuchus* + *Archosaurus* + *Sarmatosuchus*):

All trees: Char. 21: 1 --> 2; Char. 41: 0 --> 1; Char. 251: 0 --> 1;

*Proterosuchus*:

All trees: Char. 8: 2 --> 0; Char. 120: 0 --> 1;

*Archosaurus*:

All trees: Char. 39: 1 --> 0;

*Sarmatosuchus*:

All trees: Char. 98: 0 --> 1; Char. 125: 1 --> 0;

Node *Koilamasuchus* + *Fugusuchus* + *Archosauria*:

All trees: Char. 15: 2 --> 1; Char. 39: 1 --> 2; Char. 43: 0 --> 1; Char. 61: 1 --> 0; Char. 63: 1 --> 0; Char. 88: 0 --> 1; Char. 149: 2 --> 1; Char. 177: 0 --> 2; Char. 186: 0 --> 1; Char. 248: 0 --> 1;

*Fugusuchus*:

All trees: No autapomorphies;

*Koilamasuchus*:

All trees: No autapomorphies;

Node Erythrosuchidae + Archosauria:

All trees: Char. 13: 0 --> 1; Char. 38: 1 --> 0; Char. 60: 0 --> 1; Char. 79: 1 --> 0; Char. 122: 0 --> 1; Char. 125: 1 --> 0;

Node Erythrosuchidae (*Garjainia* + *Shansisuchus* + *Erythrosuchus*):

All trees: Char. 17: 1 --> 0; Char. 87: 0 --> 1; Char. 99: 0 --> 1; Char. 101: 0 --> 1; Char. 118: 1 --> 0; Char. 130: 1 --> 0; Char. 134: 1 --> 0; Char. 139: 0 --> 1; Char. 144: 0 --> 1; Char. 162: 0 --> 1; Char. 184: 0 --> 1;

*Garjainia*:

All trees: Char. 120: 0 --> 1; Char. 124: 1 --> 0;

Node *Shansisuchus* + *Erythrosuchus*:

All trees: Char. 90: 0 --> 1; Char. 96: 0 --> 1;

*Shansisuchus*:

All trees: Char. 60: 1 --> 0; Char. 186: 1 --> 0; Char. 195: 1 --> 0; Char. 221: 0 --> 1; Char. 229: 0 --> 1; Char. 241: 0 --> 1;

*Erythrosuchus*:

All trees: Char. 16: 2 --> 1.; Char. 54: 0 --> 1; Char. 82: 0 --> 1;

Node Euparkeriidae + Archosauria:

All trees: Char. 42: 0 --> 12 Char. 58: 1 --> 0; Char. 73: 1 --> 0; Char. 78: 1 --> 0; Char. 81: 2 --> 1; Char. 94: 0 --> 1; Char. 119: 2 --> 0; Char. 126: 0 --> 1; Char. 160: 0 --> 1; Char. 165: 1 --> 2; Char. 192: 0 --> 1; Char. 204: 0 --> 1;

Node Euparkeriidae (*Euparkeria* + *Osmolskina*):

All trees: Char. 9: 3 --> 0; Char. 20: 0 --> 1; Char. 47: 0 --> 1; Char. 142: 0 --> 1; Char. 168: 1 --> 0; Char. 177: 2 --> 1; Char. 223: 0 --> 1;

*Euparkeria*:

All trees: Char. 148: 1 --> 2;

*Osmolskina*:

All trees: Char. 25: 1 --> 0; Char. 29: 0 --> 1; Char. 75: 0 --> 1; Char. 251: 0 --> 1;

Node Proterochampsia + Archosauria:

All trees: Char. 6: 0 --> 1; Char. 46: 1 --> 0; Char. 65: 1 --> 2; Char. 90: 0 --> 1; Char. 93: 0 --> 1; Char. 112: 1 --> 0; Char. 117: 0 --> 1; Char. 138: 0 --> 1; Char. 140: 0 --> 1;

Some trees: Char. 15: 1 --> 0;

Node Proterochampsia (*Chanaresuchus* + Doswellidae):

All trees: Char. 9: 3 --> 1; Char. 43: 1 --> 2; Char. 114: 1 --> 0; Char. 195: 1 --> 0;

Some trees: Char. 243: 1 --> 0;

*Chanaresuchus*:

All trees: Char. 10: 0 --> 1; Char. 39: 2 --> 0; Char. 45: 0 --> 1; Char. 94: 1 --> 0; Char. 109: 1 --> 0; Char. 148: 1 --> 0; Char. 197: 1 --> 0; Char. 238: 0 --> 1; Char. 239: 0 --> 1; Char. 241: 0 --> 1; Char. 251: 0 --> 1; Char. 252: 1 --> 0;

Node Doswellidae (*Doswellia* + *Vancleavea*):

All trees: Char. 53: 0 --> 1; Char. 60: 1 --> 0; Char. 76: 1 --> 0; Char. 143: 1 --> 0; Char. 180: 0 --> 1; Char. 181: 1 --> 0; Char. 203: 1 --> 0;

*Doswellia*:

All trees: Char. 5: 1 --> 0; Char. 16: 2 --> 0; Char. 42: 2 --> 0; Char. 108: 1 --> 0; Char. 120: 0 --> 1; Char. 128: 1 --> 0; Char. 142: 0 --> 1; Char. 178: 1 --> 0; Char. 250: 0 --> 1;

*Vancleavea*:

All trees: Char. 56: 0 --> 1; Char. 58: 0 --> 1; Char. 103: 0 --> 2; Char. 177: 2 --> 0; Char. 186: 1 --> 0; Char. 207: 0 --> 1; Char. 249: 0 --> 1;

Node Archosauria (Pseudosuchia + Ornithodira):

All trees: Char. 7: 0 --> 1; Char. 127: 0 --> 1; Char. 149: 1 --> 2; Char. 224: 0 --> 1; Char. 227: 0 --> 1;

Node Pseudosuchia (*Parasuchus* + *Terrestrisuchus*):

All trees: Char. 190: 0 --> 1; Char. 216: 0 --> 1; Char. 223: 0 --> 1; Char. 225: 0 --> 1; Char. 226: 0 --> 1; Char. 228: 0 --> 1;

Some trees: Char. 56: 0 --> 1; Char. 170: 0 --> 1; Char. 215: 0 --> 1;

*Turfanosuchus*:

All trees: Char. 9: 3 --> 1; Char. 43: 1 --> 0; Char. 120: 0 --> 1; Char. 142: 0 --> 1; Char. 241: 0 --> 1; Char. 242: 1 --> 0;

Some trees: Char. 10: 0 --> 1; Char. 14: 0 --> 1; Char. 144: 1 --> 0; Char. 173: 1 --> 2; Char. 215: 1 --> 0; Char. 243: 1 --> 0; Char. 245: 1 --> 0;

*Gracilisuchus*:

All trees: Char. 20: 0 --> 1; Char. 70: 0 --> 1; Char. 108: 1 --> 0; Char. 193: 1 --> 2;

Some trees: Char. 65: 2 --> 1; Char. 209: 0 --> 1;

*Terrestrisuchus*:

All trees: Char. 39: 2 --> 1; Char. 43: 1 --> 2; Char. 55: 1 --> 2; Char. 60: 1 --> 0; Char. 109: 1 --> 0; Char. 134: 1 --> 0; Char. 168: 1 --> 0; Char. 174: 0 --> 2; Char. 176: 0 --> 1; Char. 208: 0 --> 1; Char. 251: 0 --> 1;

Some trees: Char. 10: 0 --> 1; Char. 15: 1 --> 0; Char. 42: 2 --> 1; Char. 75: 0 --> 1;

Node *Saurosuchus* + *Qianosuchus* + Stagonolepididae + *Parasuchus*:

All trees: Char. 53: 0 --> 1; Char. 212: 0 --> 1;

Some trees: Char. 66: 1 --> 0; Char. 119: 0 --> 2;

*Saurosuchus*:

All trees: Char. 14: 0 --> 1; Char. 20: 0 --> 1; Char. 35: 1 --> 0; Char. 72: 1 --> 0; Char. 89: 0 --> 1; Char. 109: 1 --> 0; Char. 118: 1 --> 0; Char. 120: 0 --> 1; Char. 130: 1 --> 0; Char. 133: 0 --> 1; Char. 134: 1 --> 0;

Some trees: Char. 15: 1 --> 0; Char. 75: 0 --> 1; Char. 148: 1 --> 0; Char. 175: 0 --> 1; Char. 193: 1 --> 2;

*Qianosuchus*:

All trees: Char. 63: 0 --> 1; Char. 155: 1 --> 0;

Some trees: Char. 47: 0 --> 1; Char. 57: 0 --> 1; Char. 119: 2 --> 1; Char. 148: 1 --> 2;

Node Stagonolepididae + *Parasuchus*:

All trees: Char. 249: 0 --> 1; Char. 250: 0 --> 1; Char. 251: 0 --> 1;

Some trees: Char. 54: 0 --> 1; Char. 96: 0 --> 1; Char. 127: 12 --> 0; Char. 174: 0 --> 1;

Stagonolepididae:

All trees: Char. 2: 0 --> 1; Char. 70: 0 --> 1; Char. 71: 1 --> 0; Char. 76: 1 --> 2; Char. 88: 1 --> 0; Char. 97: 1 --> 0; Char. 173: 1 --> 2; Char. 189: 0 --> 1; Char. 197: 1 --> 0; Char. 221: 0 --> 1; Some trees: Char. 47: 0 --> 1; Char. 148: 012 --> 0; Char. 175: 0 --> 1; Char. 193: 12 --> 2;

*Parasuchus*:

All trees: Char. 10: 0 --> 1; Char. 11: 1 --> 2; Char. 17: 1 --> 0; Char. 33: 2 --> 0; Char. 42: 2 --> 1; Char. 43: 1 --> 0; Char. 59: 1 --> 0; Char. 94: 1 --> 0; Char. 144: 1 --> 0; Char. 151: 1 --> 0; Char. 159: 1 --> 0; Char. 169: 0 --> 1; Char. 190: 1 --> 0; Char. 191: 1 --> 0; Char. 193: 12 --> 0; Char. 224: 1 --> 0; Char. 226: 1 --> 0; Char. 245: 1 --> 0;

Some trees: Char. 148: 01 --> 2;

Node Ornithodira (Pterosauromorpha + Dinosauromorpha):

All trees: Char. 97: 1 --> 0; Char. 151: 1 --> 2; Char. 168: 1 --> 0; Char. 208: 0 --> 1; Char. 219: 0 --> 1; Char. 233: 0 --> 1; Char. 235: 0 --> 1; Char. 248: 1 --> 0;

Node Pterosauromorpha (*Scleromochlus* + *Dimorphodon*):

All trees: Char. 10: 0 --> 1; Char. 104: 1 --> 0; Char. 155: 1 --> 2; Char. 237: 0 --> 1;

*Scleromochlus*:

All trees: Char. 43: 1 --> 0; Char. 156: 1 --> 0; Char. 189: 0 --> 1;

Some trees: Char. 57: 0 --> 1;

*Dimorphodon*:

All trees: Char. 11: 1 --> 2; Char. 17: 1 --> 0; Char. 21: 0 --> 1; Char. 167: 2 --> 1; Char. 198: 0 --> 1; Char. 203: 1 --> 0;

Node Dinosauromorpha (*Lagerpeton* + Dinosauriformes):

All trees: Char. 209: 0 --> 1; Char. 213: 1 --> 2; Char. 220: 0 --> 1; Char. 229: 0 --> 1; Char. 230: 0 --> 1; Char. 234: 0 --> 1; Char. 236: 0 --> 1;

Some trees: Char. 144: 0 --> 1; Char. 243: 1 --> 0;

*Lagerpeton*:

All trees: Char. 198: 0 --> 1; Char. 238: 0 --> 2; Char. 242: 1 --> 0;

Node Dinosauriformes (*Marasuchus* + Silesauridae + Dinosauria):

All trees: Char. 176: 0 --> 1; Char. 189: 0 --> 1; Char. 190: 0 --> 1; Char. 200: 0 --> 1; Char. 201: 0 --> 1; Char. 202: 0 --> 1; Char. 218: 0 --> 1; Char. 239: 0 --> 1;

*Marasuchus*:

All trees: Char. 75: 0 --> 1; Char. 148: 12 --> 0; Char. 174: 0 --> 1; Char. 219: 1 --> 0;

Node Silesauridae + Dinosauria:

All trees: Char. 133: 0 --> 1; Char. 182: 0 --> 1; Char. 194: 0 --> 1; Char. 207: 0 --> 1; Char. 210: 0 --> 1; Char. 218: 1 --> 2; Char. 222: 0 --> 1;

Node Silesauridae (*Silesaurus*):

All trees: Char. 1: 4 --> 1; Char. 134: 1 --> 0; Char. 140: 1 --> 0; Char. 240: 1 --> 0;

Node Dinosauria (*Herrerasaurus* + *Eoraptor*):

All trees: Char. 39: 2 --> 1; Char. 144: 1 --> 2; Char. 162: 0 --> 1; Char. 174: 0 --> 2; Char. 193: 1 --> 2; Char. 197: 1 --> 0; Char. 198: 0 --> 1;

*Herrerasaurus*:

All trees: Char. 45: 0 --> 1; Char. 46: 0 --> 1; Char. 52: 1 --> 0; Char. 71: 1 --> 0; Char. 106: 1 --> 0; Char. 109: 1 --> 0; Char. 130: 1 --> 0; Char. 142: 0 --> 1; Char. 170: 0 --> 1; Char. 219: 1 --> 0; Char. 247: 1 --> 0;

*Eoraptor*:

All trees: Char. 8: 2 --> 0; Char. 9: 3 --> 1; Char. 17: 1 --> 0; Char. 21: 0 --> 1; Char. 159: 2 --> 1; Char. 201: 1 --> 0;

Some trees: Char. 14: 0 --> 1; Char. 57: 0 --> 1.

**Supplementary References**

1. M. D. Ezcurra, A. Lecuona, A. Martinelli. A new basal archosauriform diapsid from the Early Triassic of Argentina. *J. Vert. Paleontol.* **30**, 1433–1450 (2010).

2. M. D. Ezcurra, T. Scheyer, R. J. Butler. The origin and early evolution of Sauria: reassessing the Permian saurian fossil record and the timing of the crocodile-lizard divergence. *PLoS ONE* **9**, e97828 (2014).

3. W. P. Maddison, D. R. Maddison. Mesquite: a modular system for evolutionary analysis. Version 3.03 http://mesquiteproject.org (2015).
